# Designable and dynamic single-walled stiff nanotubes assembled from sequence-defined peptoids

**DOI:** 10.1038/s41467-017-02059-1

**Published:** 2018-01-18

**Authors:** Haibao Jin, Yan-Huai Ding, Mingming Wang, Yang Song, Zhihao Liao, Christina J. Newcomb, Xuepeng Wu, Xian-Qiong Tang, Zheng Li, Yuehe Lin, Feng Yan, Tengyue Jian, Peng Mu, Chun-Long Chen

**Affiliations:** 1 Physical Sciences Division, Pacific Northwest National Laboratory, Richland, WA 99352 USA; 2 Institute of Rheological Mechanics, Xiangtan University, Xiangtan, Hunan 411105 China; 3 School of Mechanical and Materials Engineering, Washington State University, Pullman, WA 99164 USA; 4 Center for Biomaterials and Biopathways, Department of Chemistry, Zhejiang University, Hangzhou, Zhejiang 310027 China; 5 School of Petroleum Engineering, State Key Laboratory of Heavy Oil Processing, China University of Petroleum (East China), Qingdao, 266580 China; 6 College of Chemistry and Chemical Engineering, Linyi University, Linyi, Shandong 276005 China; 7 Department of Mechanical Engineering and Materials Science and Engineering Program, State University of New York, Binghamton, NY 13902 USA

## Abstract

Despite recent advances in the assembly of organic nanotubes, conferral of sequence-defined engineering and dynamic response characteristics to the tubules remains a challenge. Here we report a new family of highly designable and dynamic nanotubes assembled from sequence-defined peptoids through a unique “rolling-up and closure of nanosheet” mechanism. During the assembly process, amorphous spherical particles of amphiphilic peptoid oligomers crystallize to form well-defined nanosheets before folding to form single-walled nanotubes. These nanotubes undergo a pH-triggered, reversible contraction–expansion motion. By varying the number of hydrophobic residues of peptoids, we demonstrate tuning of nanotube wall thickness, diameter, and mechanical properties. Atomic force microscopy-based mechanical measurements show peptoid nanotubes are highly stiff (Young’s Modulus ~13–17 GPa). We further demonstrate the precise incorporation of functional groups within nanotubes and their applications in water decontamination and cellular adhesion and uptake. These nanotubes provide a robust platform for developing biomimetic materials tailored to specific applications.

## Abstract (web summary)

The application potential of organic nanotubes is currently limited by their lack of designable or dynamic properties. Here, Chen et al. use sequence-defined peptoids to assemble a new family of pH-responsive stiff nanotubes whose dimensions, components and functions can be easily tailored.

## Introduction

One-dimensional (1D) organic nanotubes (ONTs) have emerged as an important class of nanostructures with high aspect ratios and large internal surface areas for applications in nanotechnology and medicine, including catalysis, optics, electronics, chemical or biological sensors, and tissue engineering^1–12^. In the past decades, a wide range of single-walled and multi-walled ONTs have been synthesized through the assembly of amphiphilic small molecules or macromolecules, such as lipid- or lipid-like molecules^3,11^, synthetic polymers^5^, peptides^2,12^, proteins^2,10^, peptide-polymer hybrids^8^, and DNAs^6^. Despite all these advances in ONT development, integration of sequence-defined engineering and dynamic response characteristics into ONTs is a challenging task. In addition, despite the continuing emergence of new applications of ONTs, fundamentals, such as the mechanism of the formation of ONTs that is crucial to ONT properties and applications, are still the missing piece of the puzzle.

Peptoids (poly-N-substituted glycines) are a type of sequence-controlled molecules that were developed as attractive protein-mimetics to combine the advantages of both synthetic polymers and biopolymers^13,14^. They can be cheaply and easily synthesized and exhibit a narrow polydispersity index of molecular weight, and have large side chain diversity. Peptoids are biocompatible and show great promise for protein-like molecular recognition. Moreover, in contrast to peptides and proteins, they are highly thermally and chemically stable and offer the unique simplicity for tuning functions because they lack intra- and intermolecular backbone hydrogen bonds^13,15–18^.

Our approach to nanotube design overcomes the limitations of previously reported ONTs through the assembly of sequence-defined peptoids that contain aromatic domains, in which peptoids could offer easy tunability and the adjacent aromatic segments of peptoids could slide with respect to one another to offer externally-triggered structural dynamics. We demonstrate the assembly of amphiphilic peptoid oligomers (APOs) into a new family of highly designable, stiff, and dynamic single-walled peptoid nanotubes (SW-PNTs) through a unique “rolling-up and closure of nanosheet” mechanism. These SW-PNTs undergo a drastic contraction of ~46% in height as solution pH decreases, and this pH-triggered response is reversible. We further demonstrate that SW-PNTs can be rationally engineered to tune their surface chemistry, wall thickness, PNT diameter, and mechanical properties. By precisely introducing β-cyclodextrins (CDs) or RGD peptides within SW-PNTs, we demonstrate the applications of functional PNTs in purifying azo-contaminated water and in enhancing cancer cell adhesion and uptake. Our study offers the first route to assembly of dynamic and stiff nanotubes from sequence-defined synthetic molecules.

## Results

### Self-assembly of APOs into PNTs

In our experiments, through a previously-developed submonomer synthesis method^18,19^, we have designed and synthesized a series of APOs, Nce_6_Nbpm_*n*_ (*n* = 5, 6, 7) and their functionalized sequences, Nbpm = N-[(4-bromophenyl)methyl] glycines and [Nce = N-(2-carboxyethyl)glycine] (Fig. 1a). These APOs have a polar domain containing six Nce groups and a hydrophobic region having 5–7 Nbpm groups. The detailed synthesis and characterizations of these APOs are shown in the Methods and in the Supplementary Figs. 1–18.

**Fig. 1:**
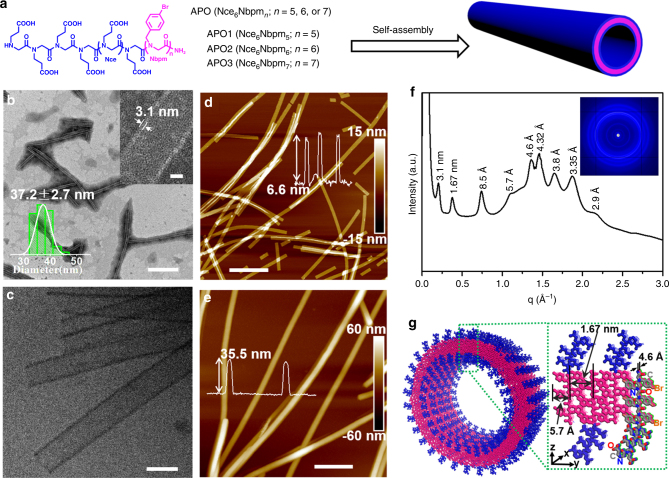
APOs assembled into highly-ordered SW-PNTs. **a** APOs structures and the scheme showing the assembly of APOs into SW-PNTs. **b** TEM image of PNTs assembled from APO2 (scale bar, 500 nm); top right corner inset is the high-resolution TEM image showing the PNT wall thickness (scale bar, 10 nm); lower left corner inset is the statistical size distribution of PNT diameter (37.2 ± 2.7 nm) obtained by analyzing 50 PNTs. **c** Cryo-TEM image of APO2-PNTs (scale bar, 50 nm). **d** Ex situ AFM height image of APO2-PNTs showing their thickness of 6.6 nm under dry condition (scale bar, 600 nm). **e** In situ AFM height image of APO2-PNTs showing the thickness of 35.5 nm in water (scale bar, 500 nm). **f** XRD spectrum of APO2-PNTs. The values above each peak is calculated according to the formula of *d* = 2*π*/*q*. **g** A proposed model showing the molecular packing of APO2 within PNTs, in which the peptoid backbone to backbone distance is 4.6 Å along the *x* direction and is 1.67 nm along the *y* direction; APO hydrophobic domains were highlighted in pink and the polar domains were in blue; one APO2 molecule was highlighted with elements in various colors (nitrogen, blue; oxygen, red; carbon, gray; bromide, orange)

For assembly of APOs into PNTs (Fig. 1a), APO solutions (5.0 mM, in water and acetonitrile (v/v = 50:50, pH 2.5–3)) were left undisturbed at 4 °C for slow crystallization. Gel-like materials containing a large amount of crystalline free-floating PNTs were formed about 2 or 3 days later from amorphous phases (Methods and Supplementary Fig. 19 for details). Negatively stained transmission electron microscopy (TEM) images showed that APO2 (Nce_6_Nbpm_6_) formed uniform nanotubes exhibiting a wall thickness of 3.1 ± 0.1 nm (top right corner inset of Fig. 1b), similar to the thickness of bilayer-like peptoid membranes we previously reported^18^. The average PNT diameter obtained from these TEM images is 37.2 ± 2.7 nm (lower left corner inset of Fig. 1b). The formation of uniform APO2-PNTs was further confirmed by cryogenic transmission electron microscopy (Cryo-TEM) images (Fig. 1c; Supplementary Fig. 20a, b), where two high-contrast dark walls and a low-contrast bright channel of the tubular structures were clearly observed without staining. The wall thickness and diameter of PNTs obtained from Cryo-TEM are ~3.1 nm and 35 nm, respectively, similar to those obtained from negatively stained TEM data. Atomic force microscopy (AFM) studies (Fig. 1d, e) were further used to characterize APO2-PNTs. Ex situ AFM (Fig. 1d) shows that they exhibit a height of ~6.6 nm, which is about 0.4 nm more than two times of the PNT wall thickness, indicating APO2-PNTs were deformed under dry conditions. The PNT height measured from in situ AFM images is around 35.5 nm (Fig. 1e), which is comparable to the diameters obtained from TEM data. These AFM results further confirmed the assembly of APO2 into PNTs. Moreover, the different PNT heights from ex situ and in situ AFM imaging suggest that APO2-PNTs are dynamic enough to deform. Both AFM and TEM data show that APO2-PNTs exhibit a length over several micrometers (Fig. 1b, d, e; Supplementary Fig. 20a–c) and sonication is an effective way to cut nanotubes in short (Supplementary Fig. 20d).

To know more information of the APO2-PNT structure, we performed synchrotron-based X-ray diffraction (XRD). XRD data demonstrate that APO2-PNTs are highly crystalline (Fig. 1f). The first low *q* peak (*d* = 3.1 nm) corresponds to the PNT wall thickness, consistent with those obtained from TEM results. The 1.67 nm spacing corresponds to the distance between two peptoid backbones in the direction of Nbpm groups facing each other (Fig. 1g). The peak at 5.7 Å is ascribed to the ordered packing of aromatic side chains within the hydrophobic segments. The strong peak at *q* = 13.6/nm is from the alignment of peptoid chains, which leads to a spacing of 4.6 Å between peptoids^18^. The peak at 3.35 Å could be ascribed to the spacing between residues along the chain direction. The presence of extensive *π*-stacking is evidenced by the peaks at 4.32, 3.8, and 2.9 Å^20^.

Interestingly, similar APO2-PNTs formed even when the crystallization solution is at pH 7.4 or 12 (Supplementary Fig. 21a). The fact that varying crystallization solution pH did not abolish PNT formation suggests that hydrophobic interactions contribute significantly to stabilization and formation of PNTs. To further demonstrate the ordering of hydrophobic domains is the key to forming tubular structures, we synthesized peptoids APO4-APO8 by only varying the residues of polar domains or changing the number of Nce groups. Among them, APO4 (Nae_3_Nce_3_Nbpm_6_) contains three Nae [Nae = N-(2-aminoethyl)glycine] and three Nce residues, APO5 (Noe_6_Nbpm_6_) contains six non-carboxyl-containing Noe6 [Noe = N-(2-hydroxylethyl)glycine] residues, APO6 (Nce_3_Nbpm_6_) contains three Nce residues, APO7 (Nce_5_Nbpm_6_) contains five Nce residues, and APO8 (Nce_9_Nbpm_6_) has nine Nce residues. As we expected, all of these peptoids assembled into PNTs whose structures are similar to those of APO2-PNTs (Supplementary Figs. 21b, c, 22), suggesting that as long as the six Nbpm residues are remained, the polar domains of these tube-forming peptoids can be varied to tune the surface chemistry of PNTs. On the other hand, when the polar domain Nce_6_ is remained, changing the hydrophobic domain Nbpm_6_ to Nbpm_4_ (for APO9: Nce_6_Nbpm_4_), Nbpm_8_ (for APO10: Nce_6_Nbpm_8_), or to Npm_6_ {for APO11: Nce_6_Npm_6_; Npm = N-[(phenyl)methyl]glycine} led to the formation of non-tubular structures (Supplementary Fig. 23), further suggesting that the ordering of hydrophobic domains is the key to forming tubular structures.

Based on the TEM, AFM, and XRD results, we proposed a molecular packing model of APO2-PNTs (Fig. 1g). In the tubular walls, the hydrophobic segments of APO2 are packed with each other and embedded in the center of the tubular wall, while the hydrophilic segments were distributed on the exterior surface of the wall, exhibiting a similar packing as we described in the formation of bilayer-like peptoid membranes^18^. The observed 3.1 nm wall thickness of APO2-PNT further supports this model showing the bilayer-like packing of APO2 to form tubular structures.

**Fig. 2:**
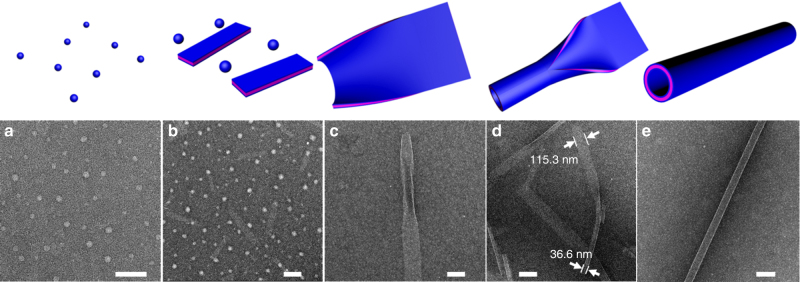
Time-dependent TEM images showing the assembly pathway of APO2-PNTs. **a** TEM image of APO2 nanospheres (~26.2 nm in diameter), which were formed immediately after APO2 was completely dissolved in the mixture of H_2_O and CH_3_CN (scale bar, 200 nm). **b** TEM image showing APO2 assembled into a mixture of nanospheres (~44.9 nm in diameter) and nanoribbons (with a width of 75–120 nm and length of 200–600 nm) after 0.5 h crystallization (scale bar, 200 nm). **c** TEM image showing one nanoribbon with partially rolled up edges after 24 h crystallization of APO2 (scale bar, 100 nm). **d** TEM image shows that APO2 formed partially converted nanotubes after 48 h crystallization (scale bar, 200 nm); the measured width of one nanoribbon (about 115.3 nm) is 3.15 (approximately equal to *π*) times the diameter of this closed nanotube (about 36.6 nm), which is in accordance with the circumference formula (*L* = *πD*). **e** TEM image showing the APO2-PNT formed after 72 h crystallization (scale bar, 100 nm)

To determine the assembly pathway, negatively stained TEM images were collected at the different stages of PNT assembly. Because PNT formation is too fast under typical assembly conditions (APO2 5.0 mM, Supplementary Fig. 24), we slowed the process by reducing the concentration to 0.5 mM to capture the PNT intermediates. As shown in Fig. 2a, APO2 formed uniform nanospheres with a diameter of 26.2 ± 5.1 nm (Supplementary Fig. 25) after they were completely dissolved in the mixture of H_2_O and CH_3_CN. After slowly evaporating the solvent over 30 min at 4 °C, TEM data showed that APO2 assembled into a mixture of nanospheres (Fig. 2b) with a diameter of 44.9 ± 7.5 nm (Supplementary Fig. 26) and nanoribbons (Fig. 2b) with a width of 75–120 nm and length of 200–600 nm. Between 1–72 h of crystallization, the ribbons began to roll up, fold and close up to form elongated SW-PNTs (Fig. 2c–e). Figure 2c and Supplementary Fig. 27 demonstrate examples where the ribbons began to roll up from the edge of the nanoribbon. Next, the rolling-up ribbon folded (Fig. 2d; Supplementary Fig. 28) and closed up (Fig. 2e) to generate SW-PNTs. Based on the TEM data (Fig. 2d), the measured width of this nanoribbon (about 115.3 nm) is 3.15 (approximately equal to *π*) times the diameter of this closed nanotube (about 36.6 nm), which is in accordance with the circumference formula (*L* = *πD*). In brief, APO2-PNTs were formed through a unique mechanism by rolling-up, folding, and closure of nanoribbons, which is different from the previously reported peptide and peptoid nanotube formations^2,17^.

### Tunability of PNT wall thickness and diameter

**Fig. 3:**
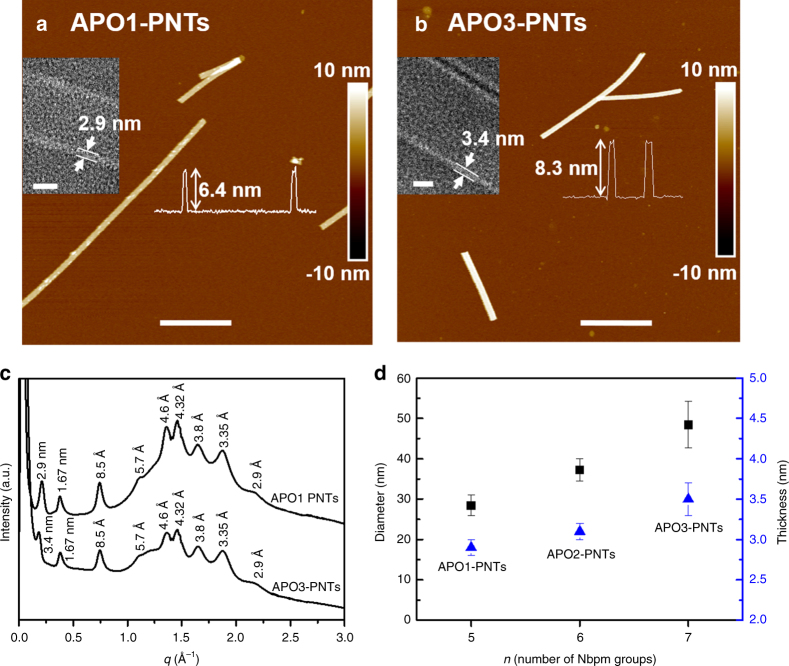
Tunability of PNT wall thickness and diameter. **a** AFM height image of APO1-PNT (scale bar, 500 nm); the left inset is the high-resolution TEM image of one APO1-PNT showing the PNT wall thickness (scale bar, 10 nm) and the right inset is the AFM height measurement showing the PNT height. **b** AFM height image of APO3-PNT (scale bar, 800 nm); the left inset is the high-resolution TEM image of one APO3-PNT showing the PNT wall thickness (scale bar, 10 nm) and the right inset is the AFM height measurement showing the PNT height. **c** XRD spectra of APO1-PNTs and APO3-PNTs, showing both APO1-PNT and APO3-PNT have similar structures. **d** PNTs wall thickness and diameter tuned by varying the number (*n*) of Nbpm side chains of APOs; Each value represents the mean standard deviation obtained from measuring at least 50 PNTs

Wall thickness and diameter are two important parameters for nanotubes^11^. Based on our proposed model that the packing of hydrophobic domains is significant for nanotube formation, we reason that PNT wall thickness and diameter can be controlled by varying the number (*n*) of Nbpm groups of tube-forming APOs. To confirm that, we further investigated the assembly of APO1 (Nce_6_Nbpm_*n*_, *n* = 5) and APO3 (Nce_6_Nbpm_*n*_, *n* = 7) (Fig. 1a). Both TEM (Supplementary Figs. 29, 30) and AFM (Fig. 3a, b) results showed that both APO1 and APO3 assembled into uniform SW-PNTs, which APO1-PNT diameter is about 28.4 ± 2.6 nm (Supplementary Fig. 29d) and APO3-PNT diameter is around 48.4 ± 5.8 nm (Supplementary Fig. 30d). The PNT wall thickness is about 2.9 ± 0.1 nm for APO1-PNTs (inset of Fig. 3a) and 3.4 ± 0.2 nm for APO3-PNTs (inset of Fig. 3b). Ex situ AFM data (inset curves of Fig. 3a, b) show that the PNT height is about 6.4 nm for APO1-PNTs and 8.3 nm for APO3-PNTs, indicating a similar PNT deformation under dry conditions as we observed in the APO2-PNT system. The PNT height observed from in situ AFM images (Supplementary Fig. 31) is 27.7 nm for APO1-PNTs and 46.8 nm for APO3-PNTs, consistent with the PNT diameters measured from TEM results. The different PNT heights under air and in water indicate that these APO1- and APO3-PNTs are all dynamic enough to deform under drying process. The powder XRD results (Fig. 3c) show that both APO1- and APO3-PNTs are highly crystalline. They have nearly the same XRD peaks with the only noticeable differences at the first low *q* peak corresponding to PNT wall thickness, which is 2.9 nm for APO1-PNTs (Fig. 3c), 3.1 nm for APO2-PNTs (Fig. 1f), and 3.4 nm for APO3-PNTs (Fig. 3c). The PNT wall thicknesses obtained from XRD data is consistent with those measured from TEM results. Additionally, the difference in the first low *q* peak of XRD data indicates that the PNT wall thickness is dependent on the number of Nbpm groups. Our results demonstrate that varying the number of Nbpm groups of APOs is an effective way to tune both PNT wall thickness and diameter (Fig. 3d). Further decreasing (*n* = 4) or increasing (*n* = 8) the number of Nbpm groups led to the formation of non-tubular structures (Supplementary Fig. 23a, b).

### PNTs are highly stable and exhibit pH-responsive structural dynamics

**Fig. 4:**
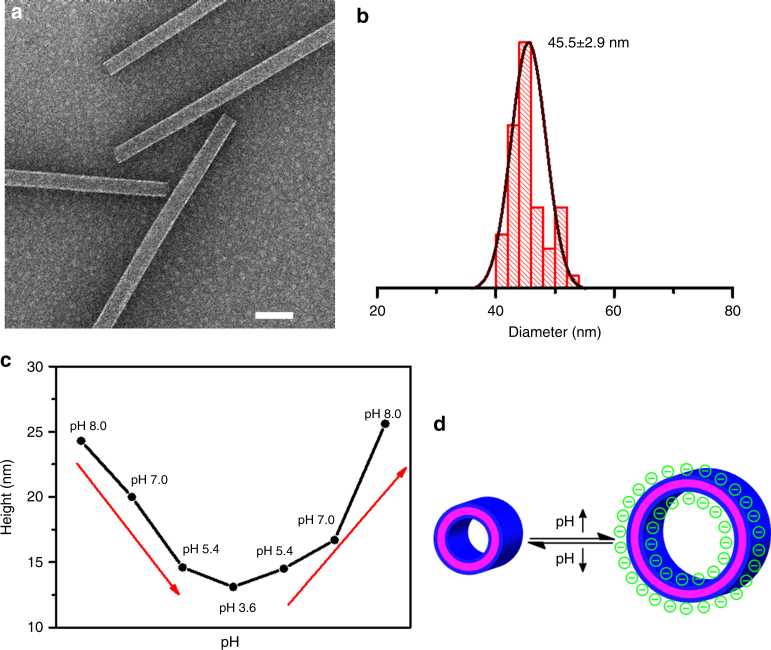
pH-responsive structural dynamics of APO2-PNTs. **a** TEM image of APO2-PNTs dispersed in pH 11.94 aqueous solution for 6 h (scale bar, 100 nm). **b** Statistical diameter distribution of pH-triggered APO2-PNTs measured from the TEM results. The number above histogram is the average tubular diameter; 50 nanotubes were analyzed for each size distribution. **c** The AFM height change of one fixed APO2-PNT in response to the solution pH variation. In the experiments, PNTs have been incubated with various aqueous solutions with different pH values (8.0, 7.0, 5.4, 3.6). The whole process was monitored in real time using in situ AFM. **d** A scheme showing the proposed mechanism of pH-triggered PNT height changes as a result of deprotonation of Nce groups

To test the stability of PNTs, we exposed them to a range of solvents as well as high temperature. As shown in Fig. 4a, APO2-PNTs survived when they were dispersed in alkaline solution (pH = 11.94) for over 6 h. The high stability of these PNTs was further demonstrated as they remained intact after being incubated in a mixture of water and CH_3_CN, or in 1× phosphate buffered saline (PBS) buffer, or in 1.0 M NaCl, or after heating to 60 °C in aqueous solution for 3 h (Supplementary Figs. 32–34). PNTs also survived when they were placed in pure organic solvents (e.g., CH_3_CN and EtOH) for over 3 h (Supplementary Figs. 35–36).

Interestingly, an analysis of TEM data of APO2-PNTs dispersed in the pH 11.94 NaOH aqueous solution for over 6 h show that these PNTs exhibit an average diameter of about 45.5 ± 2.9 nm (Fig. 4b), which is about 23% larger than those obtained (Fig. 1b). Because Nce groups of APO2 are fully deprotonated under this condition^21^, we hypothesized that the electrostatic repulsion between deprotonated Nce groups induced the increase of PNT diameter. To test our hypothesis, we further investigated the diameter changes of APO2-PNTs deposited on mica surface under various aqueous environments. In situ AFM was used to directly monitor the height changes of APO-PNTs in response to solution pH variations in the range of 3.6–8.0. Interestingly, the AFM height of one APO2-PNT gradually changed from 24.3 to 13.1 nm (about 46% contraction in height) as solution pH decreased from 8.0 to 3.6. When the pH increased again from 3.6 to 8.0, the PNT height increased accordingly (Fig. 4c, d; Supplementary Fig. 37). These results are consistent with our hypothesis showing that electrostatic repulsions between Nce groups induce the structural dynamics of PNTs. According to the acid–base titration curve of APO2 (Supplementary Fig. 38), the carboxyl groups (Nce) started to deprotonate and became negatively-charged when the solution pH was above 3.3. Thus, when the solution pH was changed from 3.6 to 8.0, we believe that the electrostatic repulsion interaction was proportionally increased, resulting in a significant increase of AFM heights of APO2-PNTs. These pH-triggered PNT height changes further demonstrate the high stability of PNTs. As far as we know, this is the first example of PNTs assembled from sequence-defined synthetic molecules that undergo responsive dynamic structural changes while remaining intact.

### Mechanical properties of PNTs

**Fig. 5:**
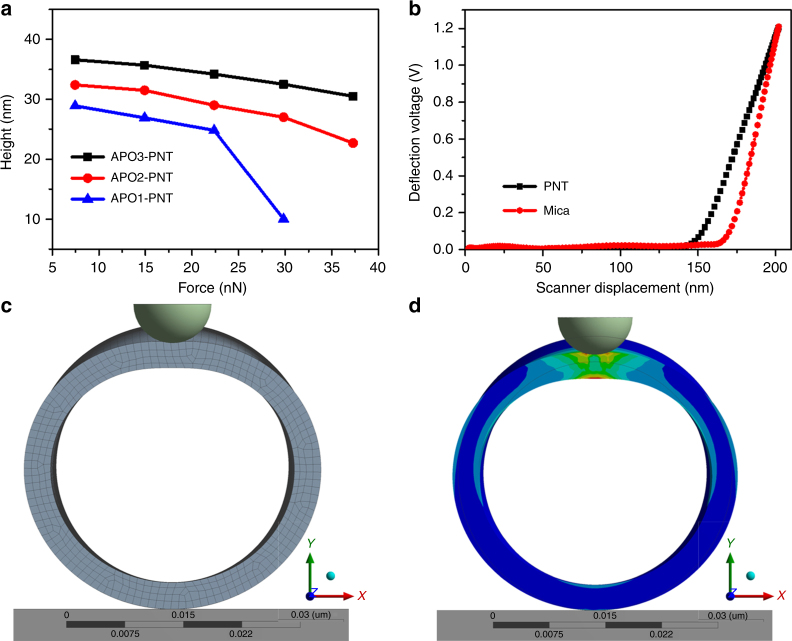
Mechanical properties of PNTs. **a** Height vs. force curves. For all experiments, the same silicon nitride cantilever (*k* = 0.4 N/m) was used. All force measurements were performed in an AFM fluid cell with deionized water. **b** A representative force vs. displacement curves. Before tapping the PNTs surface, the deflection voltage was around 0 (horizontal segment). After contacting with PNT’s surface, the deflection voltage increased gradually (slope segment). **c**, **d** FEM simulation for indentation of a PNT. The curvature radius of the indenter was set at 5 nm. The length to diameter ratio of the PNT was 10:1. **c** Before indentation. **d** After indentation

In addition to chemical and thermal stability, mechanical properties of the nanostructures play a significant role in translation for applications (e.g., nanodevices)^22^. Direct measurements of the mechanical properties of free-standing, hydrated nanostructures remains a significant challenge. Traditional macroscale tests that probe bending or tensile properties are difficult on the nanoscale^22–24^. As a new AFM technique, peak force quantitative nanomechanics (PFQNM)^25^ was utilized here to investigate the mechanical property of PNTs in liquid environments. PNTs’ stiffness and Young’s modulus can be calculated through analyzing the force vs. distance (FD) curves^26^. In our study, we used this technique to directly measure the radial strength of a single PNT. As shown in Fig. 5a, the PNT’s height gradually decreased with the augment of the top loading force. Figure 5b is a typical FD curve of nanotubes, depicting the nanotubes’ representative mechanical responses. The slope of FD curves changed abruptly when the tip reached the top of PNTs. The linear relationship is sketched between the force and displacement of PNTs even at a high setpoint of 1.2 V, further certifying the hollow structures of PNTs. PNTs assembled from APO2 and APO3 resist the stress and maintain their tubular architectures even under high load of 37 nN. When the load was removed, PNTs returned to their original shape (Supplementary Fig. 39). However, PNT assembled from APO1 collapsed and dissolved when the loading force was above 30 nN (Supplementary Fig. 40). We attribute this behavior to disruption of the APOs’ molecular packing (Fig. 1g), where hydrophobic domains break and are exposed to solvent. The APO3 with the largest number of Nbpm groups will exhibit the highest hydrophobic interactions, and resulted in the highest mechanical stiffness.

Based on the FD curves (Fig. 5a), the PNTs’ mechanical behavior can be described as linear elastic. In this framework, the deformation of PNTs was calculated by the finite element method (FEM) using a commercial software package (ANSYS 17.0), in which the PNTs, AFM tip, and mica substrate are modeled as an isotropic elastic cylinder, rigid sphere, and rigid flat plane, respectively. It is assumed that there is no initial deformation between PNTs and the mica surface. The viscoelastic effect induced by the surrounding fluid environment was neglected in this model. Figure 5c displayed the raw shape of the model PNT in finite element software. A quarter-symmetric model incorporating corresponding symmetric boundary conditions was implemented to reduce computation time. Figure 5d depicted that under the high load of the AFM tip, PNTs underwent an intense deformation. The deformation features were completely consistent with a thin-wall tube model. The whole elastic deformation model is presented in the Supplementary Movies 1 and 2. Based on these models, PNTs’ Young’s modulus are 13.24, 17.65, and 15.88 GPa for APO1-PNTs, APO2-PNTs, and APO3-PNTs, respectively. These values are comparable to that of the reported stiffest biological materials^22^. Because PNTs are highly stable and not susceptible to temperature and chemical treatments, these nanotubes offer great potential for applications in micromechanical systems and functional nanodevices.

### Incorporating functional groups into PNTs

**Fig. 6:**
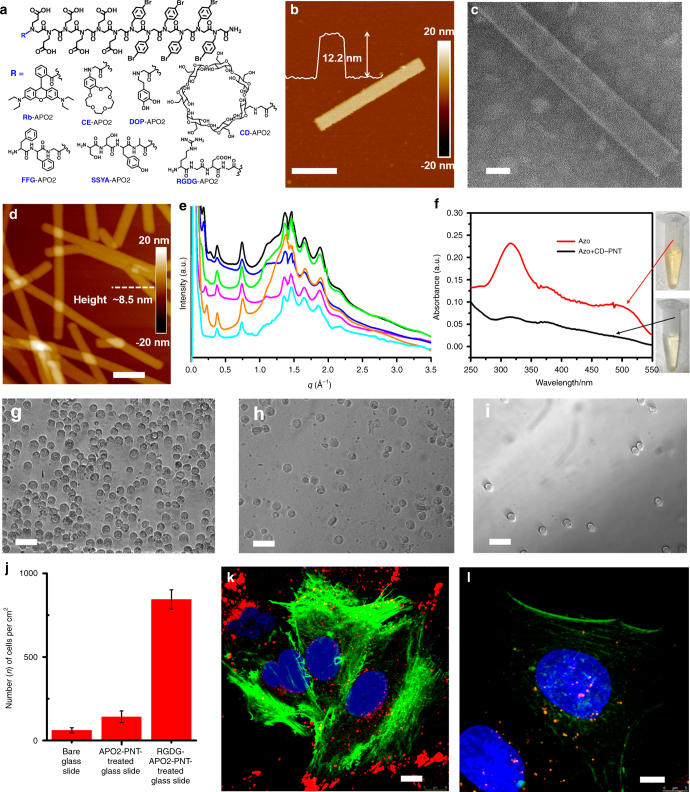
Designability and applications of PNTs. **a** Structures of seven functionalized APOs. **b** AFM height image of one PNT assembled from CD-APO2 (scale bar, 150 nm); the insert is the height measurement showing the PNT height of about 12.2 nm. **c** TEM image of one PNT assembled from CD-APO2 (scale bar, 20 nm). **d** AFM height image of PNTs assembled from RGDG-APO2 (scale bar, 200 nm). **e** X-ray diffraction (XRD) data of PNTs assembled from APO2 (magenta), CE-APO2 (blue), DOP-APO2 (black), SSYA-APO2 (green), CD-APO2 (orange), or RGDG-APO2 (cyan); all of these PNTs show similar XRD peaks, indicating they all have similar structures. **f** UV/Vis spectra showing the removal of 4-aminoazbenzene (azo) from water. Red line represents azo-contaminated water; black line represents the CD-APO2-PNT decontaminated water. **g**–**i** Optical images (scale bar, 50 μm) of A549 cancer cells absorbed on the RGDG-APO2-PNT-treated glass slide (**g**), APO2-PNT-treated glass slide (**h**), and on the bare glass slide (**i**). **j** The chart shows that the cell density of RGDG-APO2-PNT-treated glass slide is dramatically higher than those of control slides. **k**, **l** Fluorescent images showing the cellular uptake of sonication-cut-PNTs (in red color) co-assembled from APO2, RGDG-APO2, and Rb-APO2 with a molar ratio of 8:1:1 (scale bar, 8.0 μm) (**k**) and from APO2 and Rb-APO2 with a molar ratio of 9:1 (scale bar, 5.0 μm) (**l**), followed by staining with cytoskeleton actin (green) and DAPI (blue); the significantly higher density of red spots observed in Fig. 6k indicates that RGD-containing PNTs exhibited an enhanced cellular adhesion and uptake within A549 live cells

Because peptoids have unique side chain diversity and are easy to synthesize^13^. we reason that the assembly of APOs can be used as a platform to introduce functional groups within PNTs. To demonstrate this, we synthesized seven APOs each containing one of the following chemistries: a fluorescent dye Rhodamine B (Rb-APO2), crown ether (CE-APO2), biomolecule dopamine (DOP-APO2), peptides sequences (FFG-APO2, SSYA-APO2, and RGDG-APO2), or cyclic host molecule β-cyclodextrin (CD-APO2) (Fig. 6a) at the N-terminus of APO2. As expected, optical microscopy, confocal laser scanning microscopy (CLSM), TEM, and AFM characterizations show that all of these APOs assembled into PNTs (Fig. 6b, d; Supplementary Figs. 41–47); These introduced functional groups or molecules are located in both exterior and interior surfaces of the nanotubes (Supplementary Fig. 41). As shown in Fig. 6b, c and Supplementary Fig. 47a, uniform PNTs were even assembled from CD-APO2 that contains bulky CD molecules. XRD data demonstrate that PNTs assembled from functionalized APOs exhibit similar structures to those of APO2-PNTs (Fig. 6e). These results demonstrate that functional groups can be precisely incorporated into PNTs as peptoid side chains, and PNT assembly is robust to tolerate the addition of functional groups, building functional PNTs with tunable compositions and functions.

### Applications of functional PNTs

Dyes containing an azo chemical group (azo-dyes) have been widely used as textile colorants and now become one of the major toxic pollutants in water^27,28^. Among various strategies that have been developed to remove azo-dyes, physical adsorption is considered to be superior to others due to its high efficiency, ease of operation and low cost. Because CD is known to encapsulate azo-dyes, especially aromatics, through specific host–guest interactions^29,30^, and the nanotubular structure offers large surface area and high porosity^28^, assembled CD-APO2-PNTs were used here as adsorbents for the removal of azo-dyes from water, in which aromatic 4-aminoazobenzene was used as a model azo dye molecule. As shown in Fig. 6f and Supplementary Fig. 47, CD-APO2-PNTs removed the majority of 4-aminoazobenzene molecules from water within 1 h, which is evidenced by the disappearing of solution color and the significant decrease of UV/Vis absorption peak around 316 nm. As a result of this decontamination process, CD-APO2-PNTs turned into orange (Supplementary Fig. 47c) while PNTs remained intact (Supplementary Fig. 48).

On the other hand, because peptoids are biocompatible and exhibit protein-like molecular recognition^13^, we expect that these highly stable, stiff, and dynamic PNTs offer a brand new platform for biological applications. To demonstrate that, we decorated glass slides with and without RGDG-APO2-PNTs, because it is known that having RGD peptide sequences promotes the cell adhesion^31^. As shown in Fig. 6g, h, i, the glass slide coated with RGDG-APO2-PNTs exhibited the most significant adhesion of A549 cancer cells in contrast to the control slides (Fig. 6j). Such promoted cell adhesion induced by RGD-containing PNTs was also observed during the uptake of sonication-cut-PNTs within A549 live cells. As shown in Fig. 6k, l, PNTs co-assembled from APO2, RGDG-APO2, and Rb-APO2 with a molar ratio of 8:1:1 (Supplementary Fig. 49a, b) exhibited a much higher cellular adhesion and uptake (Fig. 6k), comparing to those co-assembled from APO2 and Rb-APO2 with a molar ratio of 9:1 (Supplementary Fig. 49c, d). These water decontamination, and cellular adhesion and uptake results indicate that SW-PNTs can be easily functionalized and tailored for specific applications.

## Discussion

We have reported the assembly of highly stable, stiff, dynamic, and designable PNTs from APOs. Time-dependent TEM studies show that these PNTs were formed through a unique folding mechanism. The whole assembly process includes four successive steps: amorphous spherical particles, formation of ribbon-like structures, rolling-up and folding, and closure to form nanotubes. We further demonstrated the tuning of PNTs wall thickness and diameter by varying the length of hydrophobic segments. By in situ AFM observation of one single PNT under variable pH, we demonstrated the pH-triggered reversible PNT height changes. AFM-based mechanical measurements demonstrated that these PNTs are highly stiff, exhibiting an elastic modulus in the range of 13–17 GPa, which is comparable to the stiffest known peptide nanotubes. The easy synthesis and large side chain diversity of peptoids enabled us to introduce a wide range of functional groups, such as fluorescent dye, macrocyclic compound, biomolecule, or peptides within PNTs. Because peptoids are sequence-defined, highly stable, biocompatible and exhibit protein-like selectivity for molecular recognition^13^, we expect this new type of dynamic SW-PNTs can provide a robust platform for development of biomimetic materials tailored to applications in water filtration, drug delivery, molecular sensing, biological imaging, and nanoelectronics.

## Methods

### Materials

*β*-alanine *t*-butyl ester hydrochloride was purchased from Chem-Impex International, Inc. The hydrochloride was deprotected by a sodium hydroxide aqueous solution, then extracted with CH_2_Cl_2_, filtered, and rotary evaporated for further use. *N*,*N*ʹ-diisopropylcarbodiimide, bromoacetic acid, and trifluoroacetic acid (TFA) were purchased from Chem-Impex International, Inc and used as received. 4-Bromobenzylamine was purchased from Oakwood Products, Inc and used as received. Rhodamine B, *N,N*-diisopropylethylamine (DIPEA), triisopropylsilane, and dopamine hydrochloride were purchased from Sigma-Aldrich and used as received. Tert-Butyl *N-*(2-aminoethyl)carbamate was purchased from CNH Technologies, Inc and used as received. 4ʹ-aminobenzo-15-crown 5-ether was purchased from TCI America and used as received. Fmoc-Gly-OH, Fmoc-Ala-OH, Fmoc-Ser(tBu)-OH, Fmoc-Tyr-OH, Fmoc-Phe-OH, Fmoc-Asp(OtBu)-OH, Fmoc-Arg(Pbf)-OH, and HBTU were purchased from the Aapptec and used as received. Mono-6-amino-6-deoxy-cyclodextrin (CD-NH_2_) was synthesized according to the reported literature^18^. All other amine submonomers and other reagents are obtained from commercial sources and used without further purification. MilliQ water at 18 MΩ cm was used for all experiments.

### Synthesis of amphiphilic peptoid oligomers

Some peptoids were synthesized on a commercial Aapptec Apex 396 robotic synthesizer using a solid-phase submonomer cycle as described previously^18,19^. Rink amide resin (0.09 mmol) was used to generate C-terminal amide peptoids. In this method, the Fmoc group on the resin was deprotected by adding 2 mL of 20% (v/v) 4-methylpiperidine/*N*,*N*-dimethylformamide (DMF), agitating for 20 min, draining, and washing with DMF. All DMF washes consisted of the addition of 1.5 mL of DMF, followed by agitation for 1 min (repeated five times). An acylation reaction was then performed on the amino resin by the addition of 1.6 mL of 0.6 M bromoacetic acid in DMF, followed by 0.35 mL of 50% (v/v) *N*,*N*-diisopropylcarbodiimide (DIC)/DMF. The mixture was agitated for 30 min at room temperature, drained, and washed with DMF for five times. Nucleophilic displacement of the bromide with various primary amines occurred by a 1.6 mL addition of the primary amine monomer as a 0.6 M solution in *N*-methyl-2-pyrrolidone (NMP), followed by agitation for 60 min at room temperature. The monomer solution was drained from the resin, and the resin was washed with DMF for five times. The acylation and displacement steps were repeated until a polypeptoid of the desired length was synthesized.

For manual solid-phase synthesis, rink amide resin (0.09 mmol) was used to synthesize C-terminal amphiphilic peptoids. In the synthesis procedure, the Fmoc groups on the resin were deprotected by adding 2 mL of 20% (v/v) 4-methylpiperidine/DMF, agitating for 40 min, filtering, and washing with DMF. For all DMF washes, 1 mL DMF was added and then agitated for 1 min (repeated five times). An acylation reaction was then performed on the amino resin by the addition of 1.5 mL of 0.6 M bromoacetic acid in DMF, followed by adding 0.30 mL of 50% (v/v) DIC/DMF. The mixture was agitated for 10 min at room temperature, filtered and washed with DMF for five times. Nucleophilic displacement of the bromine with different primary amines occurred by the addition of 1.5 mL of 0.6 M primary amine monomer in NMP, followed by the agitation for 10 min at room temperature. The monomer solution was filtered from the resin, and washed with DMF for five times. The acylation and displacement steps were repeated until the targeted amphiphilic peptoid was synthesized.

### Peptoid cleavage and HPLC purification

The final crude product was cleaved from the resin by addition of 95% trifluoroacetic acid (TFA) in water, which was then evaporated off under a stream of N_2_ gas. Finally, crude peptoids were dissolved in H_2_O/CH_3_CN (v/v = 1:1) for HPLC purification. The crude products were purified by reverse-phase HPLC on a XBridge Prep C18 10 μm OBDTM (10 μm, 19 mm × 100 mm), using adaptable gradient of acetonitrile in H_2_O with 0.1% TFA over 15 min. Purified peptoids were analyzed using Waters ACQUITY reverse-phase UPLC (corresponding gradient at 0.4 mL/min over 7 min at 40 °C with a ACQUITYBEH C18, 1.7 μm, 2.1 mm × 50 mm column) that was connected with a Waters SQD2 mass spectrometry system. The final peptoid product was lyophilized at least twice from its solution in a mixture (v/v = 1:1) of water and acetonitrile. The peptoid powder was finally divided into small portions (2.0 × 10^−6^ mol) and stored at −80 °C.

### Synthesis of rhodamine B-functionalized APO2

After the stepwise synthesis of APO2, 2 mL of DMF solution of rhodamine B (0.9 mmol) and 0.50 mL of 50% (v/v) DIC/DMF were added, followed by the agitation for overnight at room temperature. The monomer solution was filtered from the resin, and washed with DMF for five times.

### Synthesis of crown ether-functionalized APO2

After the stepwise synthesis of APO2, the acylation reaction was performed by the addition of 1.5 mL of 0.6 M bromoacetic acid in DMF, followed by adding 0.30 mL of 50% (v/v) DIC/DMF. The mixture was agitated for 10 min at room temperature, filtered and washed with DMF for five times. In the nucleophilic displacement step, 1.5 mL of 0.6 M 4ʹ-aminobenzo-15-crown 5-ether in NMP and tetrabutylammonium iodide (TBAI, 100 mg, 0.27 mmol) were added, followed by the agitation for 2 days at 40 °C. The monomer solution was filtered from the resin, washed with deionized water for five times, and then washed with DMF for five times.

### Synthesis of dopamine-functionalized APO2

After the stepwise synthesis of APO2, the acylation reaction was performed by the addition of 1.5 mL of 0.6 M bromoacetic acid in DMF, followed by adding 0.30 mL of 50% (v/v) DIC/DMF. The mixture was agitated for 10 min at room temperature, filtered and washed with DMF for five times. In the nucleophilic displacement step, 3 mL of DMF solution of dopamine hydrochloride (0.9 mmol) and DIPEA (0.9 mmol) was added, followed by the agitation for 30 min at room temperature. The monomer solution was filtered from the resin, and washed with DMF for five times.

### Synthesis of cyclodextrin-functionalized APO2

After the stepwise synthesis of APO2, the acylation reaction was performed by the addition of 1.5 mL of 0.6 M bromoacetic acid in DMF, followed by adding 0.30 mL of 50% (v/v) DIC/DMF. The mixture was agitated for 10 min at room temperature, filtered and washed with DMF for five times. In the nucleophilic displacement step, 1.5 mL of 0.3 M CD-NH_2_ in DMF and K_2_CO_3_ (100 mg, 0.72 mmol) were added, followed by the agitation for 3 days at 40 °C. The monomer solution was filtered from the resin, washed with deionized water for five times, and then washed well with DMF.

### Synthesis of peptide-functionalized APO2

SSYA-, FFG-, and RGDG-modified APO2s were synthesized using the same method. A detailed synthesis of SSYA-functionalized APO2 was described below:

### Synthesis of SSYA-functionalized APO2

After the stepwise synthesis of APO2, 1.6 mL of 0.6 M Fmoc-Ala-OH in NMP, 1.6 mL of 0.6 M HBTU in DMF, and 0.75 mL of DIPEA were added. The mixture was agitated for 6 h at room temperature, filtered and washed with DMF for five times. In the deprotected process, 3 mL of 20% (v/v) 4-methylpiperidine/DMF were added, agitation for 1 h. The mixture was filtered and washed with DMF for five times; the obtaining resins were then used for the next step synthesis.

1.6 mL of 0.6 M Fmoc-Tyr-OH in NMP, 1.6 mL of 0.6 M HBTU in DMF, and 0.75 mL of DIPEA were added. The mixture was agitated for 6 h at room temperature, filtered and washed with DMF for five times. In the deprotected process, 3 mL of 20% (v/v) 4-methylpiperidine/DMF were added, agitation for 1 h. The mixture was filtered and washed with DMF for five times; the obtaining resins were then used for the next step synthesis.

1.6 mL of 0.6 M Fmoc-Ser(tBu)-OH in NMP, 1.6 mL of 0.6 M HBTU in DMF, and 0.75 mL of DIPEA were added. The mixture was agitated for 6 h at room temperature, filtered and washed with DMF for five times. In the deprotected process, 3 mL of 20% (v/v) 4-methylpiperidine/DMF was added, followed by agitation for 1 h. The mixture was filtered and washed with DMF for five times; the obtaining resins were then used for the next step synthesis.

1.6 mL of 0.6 M Fmoc-Ser(tBu)-OH in NMP, 1.6 mL of 0.6 M HBTU in DMF, and 0.75 mL of DIPEA were added. The mixture was agitated for 6 h at room temperature, filtered and washed with DMF for five times. In the deprotected process, 3 mL of 20% (v/v) 4-methylpiperidine/DMF was added, followed by agitation for 1 h. The mixture was filtered and washed with DMF for five times to obtain resins containing the final crude product.

The final crude product was cleaved from the resin by addition of a mixture of 90% TFA, 5% triisopropylsilane, and 5 % water.

### Assembly of APOs into peptoid nanotubes

2.0 μmol of lyophilized peptoids powders were dissolved in 400 μL of water and acetonitrile (v/v = 1:1) mixture to make 5.0 mM clear solution, pH ranging from 2.5 to 3.0. The mixture was then put in the 4 °C refrigerator for slow evaporation. About 2 or 3 days later, gel-like materials including a large number of 1D nanotubes were obtained.

For testing the hypothesis that the ordering of hydrophobic domains is the key to forming nanotube structures, 2 μmol of lyophilized APO2 powders were dissolved in the mixture of water and acetonitrile (v/v = 1:1), and 2.0 M aqueous NaOH was used to adjust the solution to pH 7.4 or 12. The resulting 5.0 mM clear solution was then used for slow crystallization at 4 °C.

For co-assembly, a mixture of two or three APO sequences in corresponding molar ratio was dissolved in the mixture of water and acetonitrile (v/v = 1:1) to get the final peptoid concentration of 5.0 mM, the resulting clear solution was then used for slow crystallization at 4 °C. About 2 or 3 days later, gel-like materials including a large number of 1D nanotubes were obtained.

### PNT stability test

10 μL of assembled PNTs was added into 200 μL of various solutions, which include a mixture of water and acetonitrile, 1× PBS buffer, pure acetonitrile, or pure ethanol. The mixtures were incubated for 3 h. For thermal stability test, 100 μL aqueous suspension of assembled PNTs were incubated in the oven under 60 °C for 3 h. For testing the stability of PNTs in the basic condition, 10 μL of gel-like materials of PNTs was diluted with 200 μL of NaOH aqueous solution (pH 11.94), and then incubated for 6 h.

### Application of CD-APO2-PNTs in water decontamination

For decontamination test, 4-aminoazobenzene was first dissolved in acid water (pH 4.0) to make a azo-contaminated water with an azo concentration of 0.04 mmol/L, then 150 μL of 10.0 mM CD-APO2-PNTs were added into 400 μL of 4-aminoazobenzene aqueous solution, followed by incubation for 1 h. UV/Vis test was performed after the centrifugation.

### Transmission electron microscopy

TEM was performed on a FEI Tecnai instrument operating at an accelerating voltage of 200 keV. For TEM measurement, a 2 μL drop of the assembly solution was diluted in 5 μL of deionized water and put onto carbon-coated copper grids for 10 min. The droplet was dried by filter paper. In the negative staining, 5 μL of phosphotungstic acid (wt 2%) was dropped onto the TEM grid for 2 min. Finally the droplet was dried by filter paper.

### Cryogenic transmission electron microscopy

A small volume (4–5 µL) of PNT solution was deposited on a 300 mesh copper TEM grid with a lacey carbon support film (Electron Microscopy Sciences Hatfield, PA) in the humidified atmosphere of an FEI Vitrobot Mark III (FEI, Hillsboro, OR). The excess volume was blotted with filter paper and the sample was quickly plunge frozen into a reservoir of liquid ethane that was cooled by liquid nitrogen. Samples were transferred under liquid nitrogen using a Gatan 626 cryogenic holder. Samples were imaged with an FEI Tecnai T-12 fitted with a LaB6 filament operating at an accelerating voltage of 120 kV. Images were collected using a Gatan 2×2K Ultrascan 1000 charge coupled device with a “U” scintillator using Gatan Digital micrograph software.

### Atomic force microscopy

Both ex situ (in air) and in situ (in fluid) AFM tests were performed on a Bruker MultiMode 8 by using tapping mode or ScanAsyst mode at room temperature. For ex situ AFM measurements, a 2 μL drop of the assembly solution was diluted with deionized water and placed onto a freshly cleaved mica substrate for 2 min. The solution was dried by filter paper and N_2_ flow. In situ AFM measurements were performed to test the stability and height of PNTs. For stability test, PNTs were incubated with water in AFM fluid cell, in situ AFM images at different time points were collected. PNT stability in acetonitrile was tested similarly by injecting solvents into AFM fluid cells or by fully exchanging these solvents with pre-injected waters in AFM fluid cells. PNT heights in deionized water were tested similarly by incubating PNTs with deionized water in AFM fluid cell. pH-responsive structural changes of PNTs were investigated by incubating PNTs in the aqueous solutions with various pH values (3.6, 5.4, 7.0, and 8.0). For in situ AFM imaging, the solution pH decreased from 8.0 to 3.6 firstly and then retrieved from 3.6 to 8.0. (Note: Due to the electronic repulsion between PNTs and mica surfaces when carboxyl group on the surfaces of PNTs are deprotonated, we found that this type of pH-responsive PNT structural change experiments are hard to do without fixing PNTs on mica surfaces. Therefore, here we used one fixed PNT, which has a diameter of 26 nm to do this pH-responsive structural change experiment using in situ AFM).

### AFM tests for PNT mechanical properties

For the force measurements, AFM tests were performed on a Bruker MultiMode 8 by using PFQNM mode at room temperature. The spring constant (*k*, N/m) of the cantilever was measured by thermal tune method. Sensitivity (*S*, nm/V) of the cantilever, as the cantilever deflection signal vs. the voltage applied to the Z-Piezo, was calibrated on a sapphire surface. The tip radius was evaluated by testing on the standard PDMS sample. The force applied on the sample can be defined as:

1 $${F} = {k} \times {S} \times \Delta {Z}$$

Where ∆*Z*(*V*) is the amplitude of cantilever bending.

### X-ray powder diffraction

Powder XRD data were collected at a multiple-wavelength anomalous diffraction and monochromatic macromolecular crystallography beamline, 8.3.1, at the Advanced Light Source located at Lawrence Berkeley National Laboratory. Beamline 8.3.1 has a 5T single pole superbend source with an energy range of 5–17 keV. Data were collected with a 3 × 3 CCD array (ADSC Q315r) detector at a wavelength of 1.1159 Å. Data sets were collected with the detector 200 mm from the sample. PNT suspensions or pellets were pipetted onto a Kapton mesh (MiTeGen) and dry. All XRD Data were processed with custom Python scripts.

### Confocal laser scanning microscopy

CLSM was performed on the Zeiss LSM710 confocal microscope. In our experiments, PNTs were diluted in the deionized water for 10 times. For the microscope observation, the diluted aqueous solution was dropped onto glass slide, covered with coverslip, and then observed directly under the microscope.

### Preparation of PNT-coated glass slides

Glass slides were incubated with APO2-PNTs or GRDG-APO2-PNTs (100 µM) for 2 h at room temperature. Then, these slides were washed with cold PBS for three times. The obtained PNT-coated glass slides were dried and stored at 4 °C for further use.

### Cancer cell adhesion

A549 cancer cells at a cell density of 1.0 × 10^4^ per mL were incubated in 96-well plates (with bare glass slide, APO2-PNT-treated glass slide, and RGDG-APO2-PNT-treated glass slide) for 30 min. Then the medium was discarded and the glasses were washed with cold PBS three times. After that, these glasses were fixed with 4% paraformaldehyde (PFA) for 30 min at room temperature. Then the cells were treated with PBS containing 0.1% Triton X-100 for 10 min and followed with imaging and counting.

### CLSM studies of uptake of functional PNTs

The uptake of PNTs by A549 cancer cells was investigated using CLSM. A549 cell (1 × 10^5^ per mL) was used to seed glass-bottom Petri dishes and cultured for 24 h at 37 °C in an incubator. Two types of functional PNTs were used for cellular uptake: (1) PNTs co-assembled from APO2, Rb-APO2, and RGDG-APO2 with a molar ratio of 8:1:1 and 2 PNTs co-assembled from APO2 and Rb-APO2 with a molar ratio of 9:1. Sonication-cut PNTs (10 µM) were then added to the cells and further incubated for 4 h. Subsequently, the cancer cells were washed three times with cold PBS (pH 7.4) and then incubated with CellLight Actin-GFP and hoechst 33342 for other 30 min. After that, these cells were fixed with 4% PFA for 30 min. After washed with PBS for three times, the cells were imaged using CLSM.

### Data availability

All relevant data are available from the authors.

## Electronic supplementary material

Supplementary Information

Description of Additional Supplementary Information

Supplementary Movie 1

Supplementary Movie 2
