# Supplementary material for: Designable and dynamic single-walled stiff nanotubes assembled from sequence-defined peptoids
Source: Nat Commun. 2018 Jan 18;9:270. doi: 10.1038/s41467-017-02059-1 (PMC5773689; doi:10.1038/s41467-017-02059-1)
Supplement: Supplementary file 1 — Supplementary Information [file 41467_2017_2059_MOESM1_ESM.pdf]

Supplementary Figures

APO1 [Nce_6Nbpm_5]: 1922.09 (Molecular Weight), 1923.60 (Found:$[M+H]^+$)

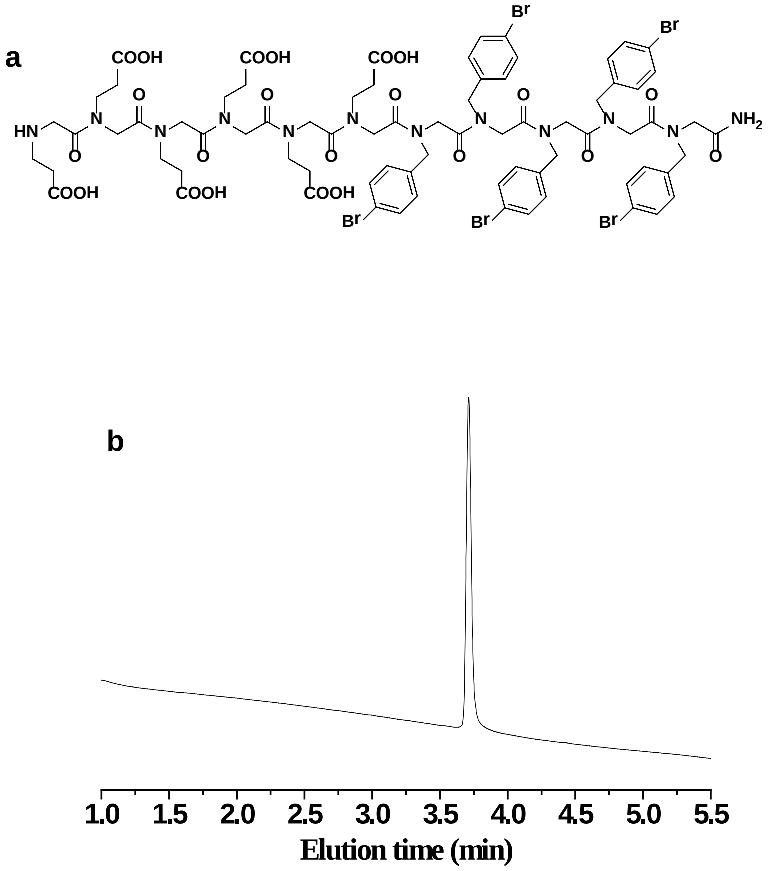

Supplementary Figure 1. Structure of Nce_6Nbpm_5 (**APO1**) (**a**) and UPLC spectrum of APO1 with the gradient of 5~95% CH_3CN in H_2O (**b**).

APO2 [Nce_6Nbpm_6]: 2148.16 (Molecular Weight), 2149.48 (Found:$[M+H]^+$)

a

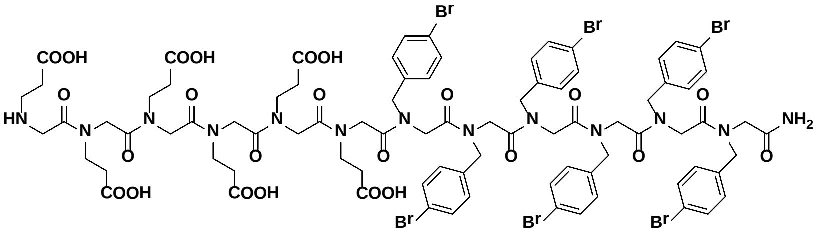

b

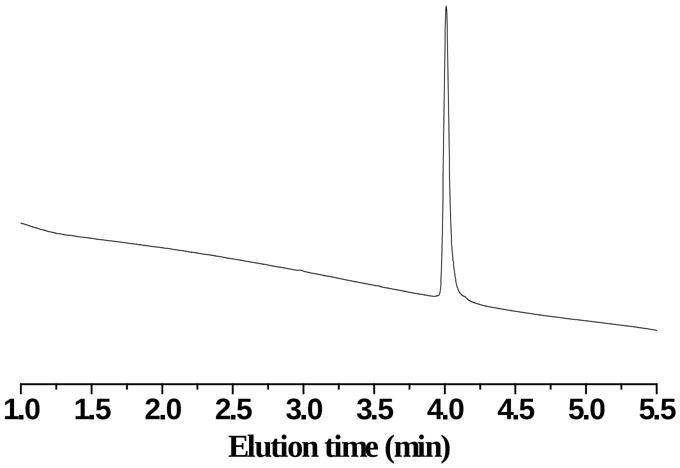

Supplementary Figure 2. Structure of Nce_6Nbpm_6 (**APO2**) (**a**) and UPLC spectrum of APO2 with the gradient of 5~95% CH_3CN in H_2O (**b**).

APO3 [Nce$_6$Nbpm$_7$]: 2374.23 (Molecular Weight), 1188.89 (Found:[M/2+H]$^+$)

a

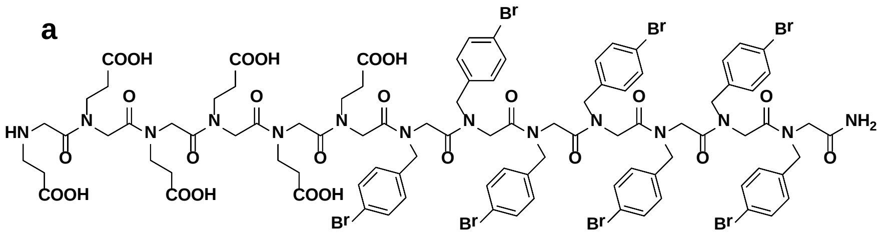

b

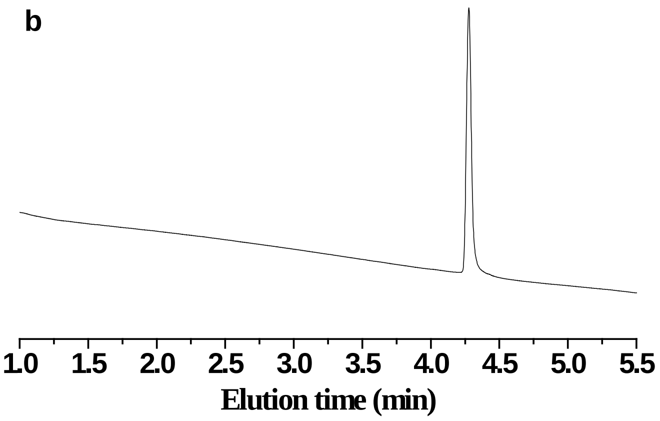

Supplementary Figure 3. Structure of Nce$_6$Nbpm$_7$ (**APO3**) (**a**) and UPLC spectrum of APO3 with the gradient of 5~95% CH_3CN in H_2O (**b**).

APO4 [$Nae_3Nce_3Nbpm_6$]: 2061.18 (Molecular Weight), 2062.91 (Found:$[M+H]^+$) and 1031.51 (Found:$[M/2+H]^+$)

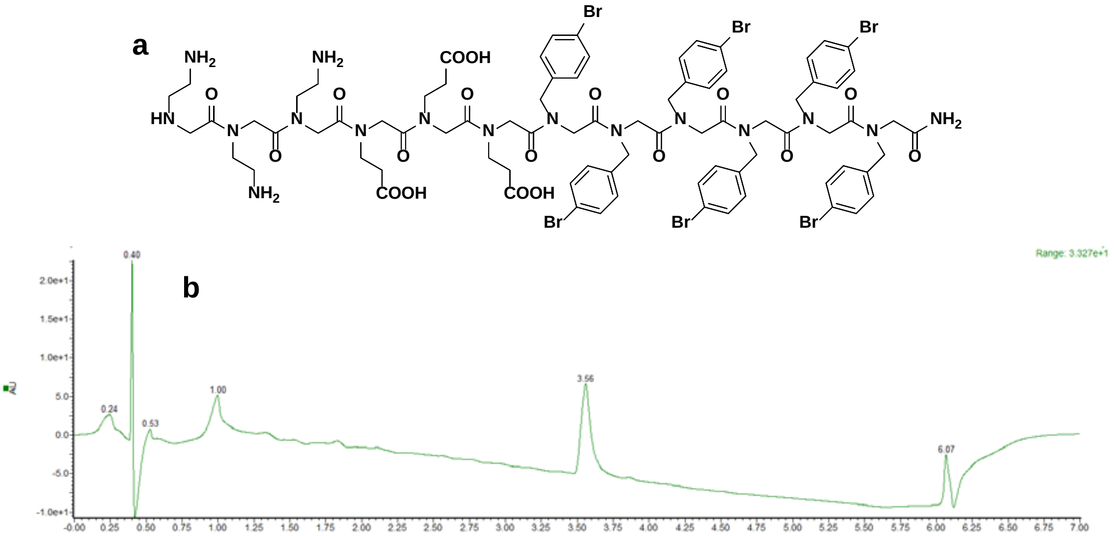

Supplementary Figure 4. Structure of $Nae_3Nce_3Nbpm_6$ (**APO4**) (**a**) and UPLC spectrum of APO4 with the gradient of 5~95% CH_3CN in H_2O (**b**).

APO5 [Noe_6Nbpm_6]: 1980.10 (Molecular Weight), 1980.61 (Found:$[M]^+$) and 990.47 (Found:$[M/2]^+$)

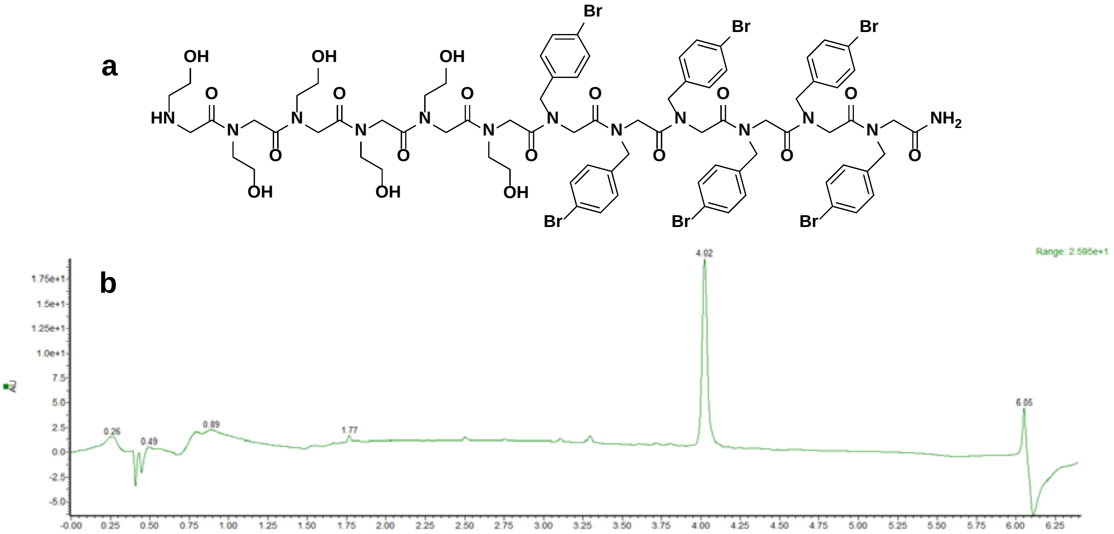

Supplementary Figure 5. Structure of Noe_6Nbpm_6 (**APO5**) (**a**) and UPLC spectrum of APO5 with the gradient of 5~95% CH_3CN in H_2O (**b**).

APO6 [Nce_3Nbpm_6]: 1760.81 (Molecular Weight), 1760.73 (Found:[M]$^+$), 880.27 (Found:[M/2]$^+$),

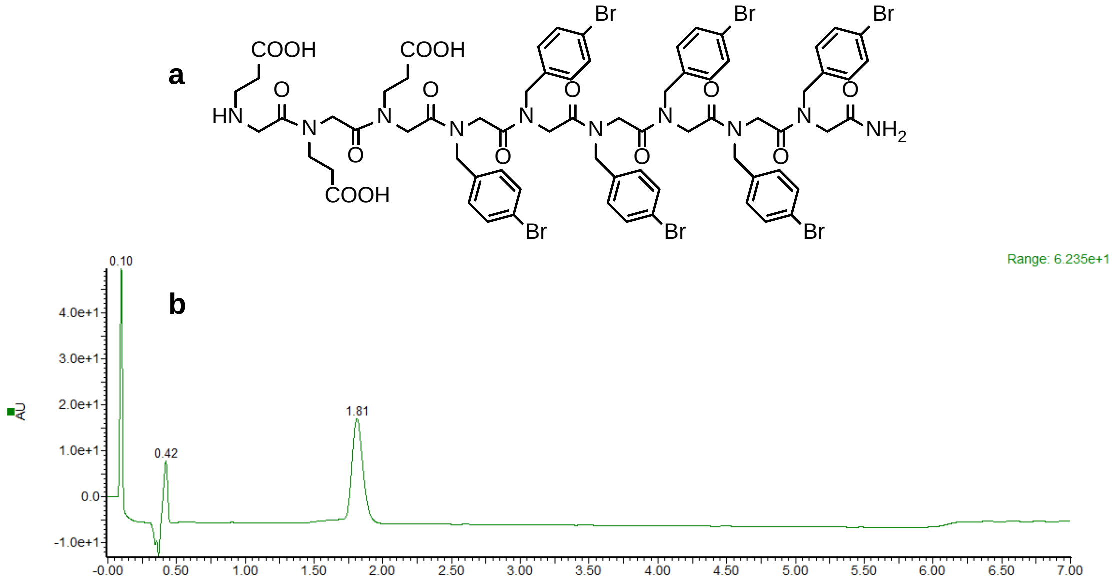

Supplementary Figure 6. Structure of Nce_3Nbpm_6 (**APO6**) (**a**) and UPLC spectrum of APO6 with the gradient of 60~70% CH_3CN in H_2O (**b**).

APO7 [Nce_5Nbpm_6]: 2019.04 (Molecular Weight), 2019.55 (Found:[M]$^+$), 1009.92 (Found:[M/2]$^+$),

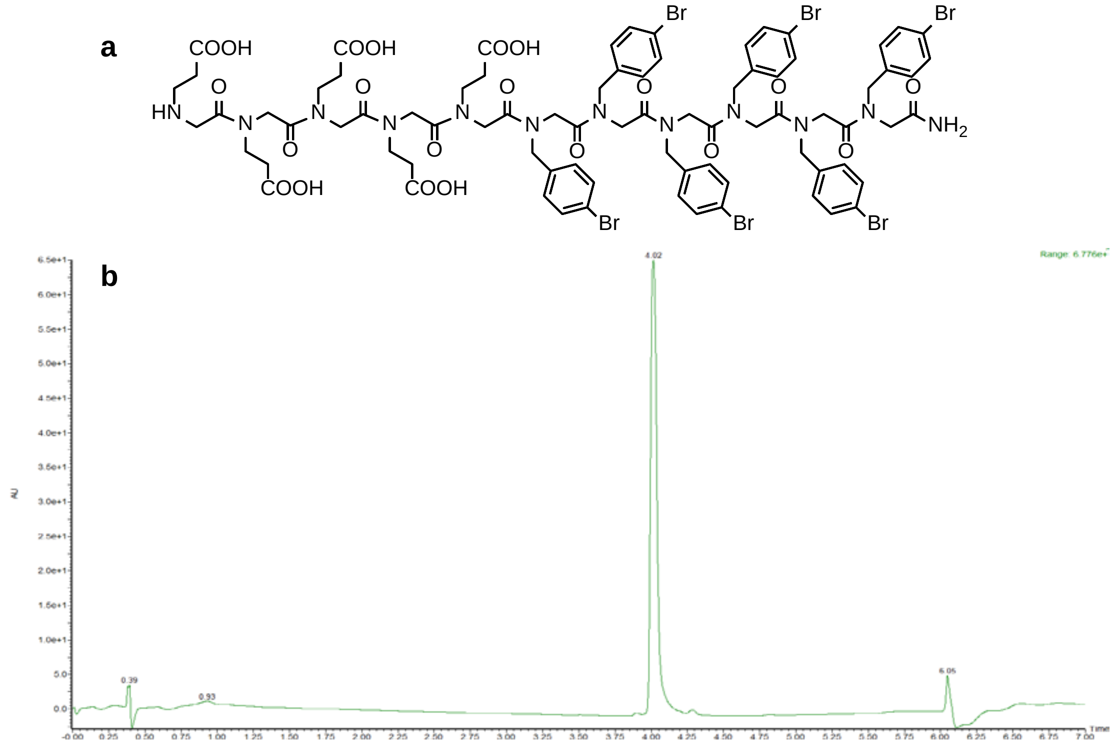

Supplementary Figure 7. Structure of Nce_5Nbpm_6 (**APO7**) (**a**) and UPLC spectrum of APO7 with the gradient of 5~95% CH_3CN in H_2O (**b**).

APO8 [Nce_9Nbpm_6]: 2535.5 (Molecular Weight), 2535.42 (Found:$[M]^+$), 1267.98 (Found:$[M/2]^+$),

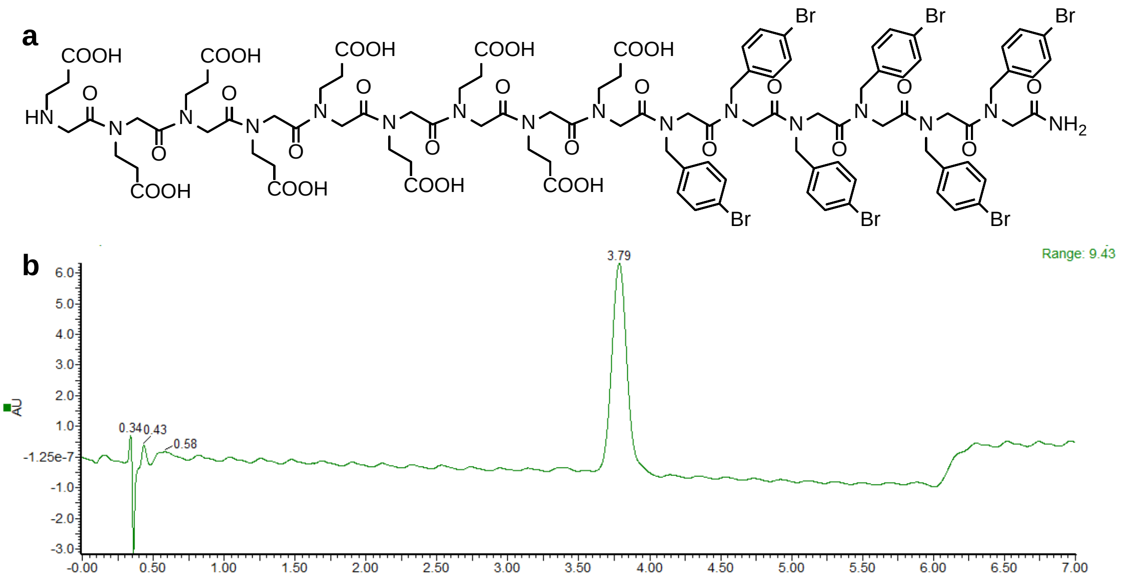

Supplementary Figure 8. Structure of Nce_3Nbpm_6 (**APO8**) (**a**) and UPLC spectrum of APO8 with the gradient of 50~60% CH_3CN in H_2O (**b**).

APO9 [Nce_6Nbpm_4]: 1696.01 (Molecular Weight), 1696.22 (Found:$[M]^+$), 839.8 (Found:$[M/2]^+$)

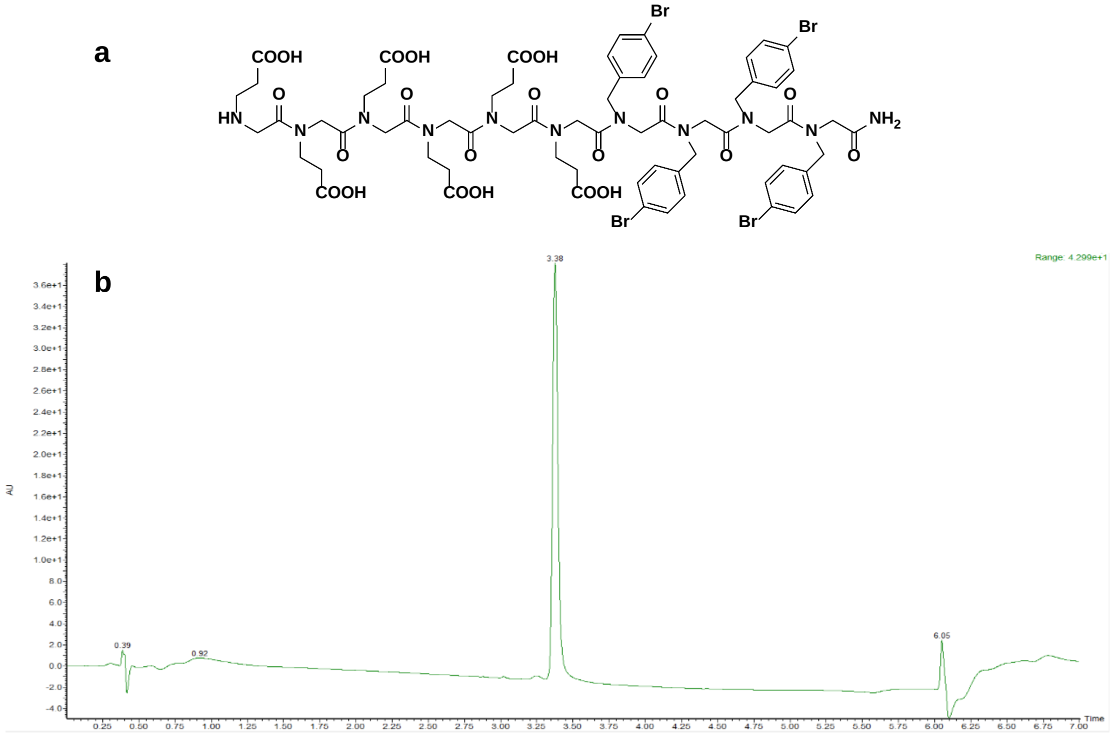

Supplementary Figure 9. Structure of Nce_6Nbpm_4 (**APO9**) (**a**) and UPLC spectrum of APO9 with the gradient of 5~95% CH_3CN in H_2O (**b**).

APO10 [Nce_6Nbpm_8]: 2600.30 (Molecular Weight), 2600.94 (Found:[M]$^+$), 1300.44 (Found:[M/2]$^+$)

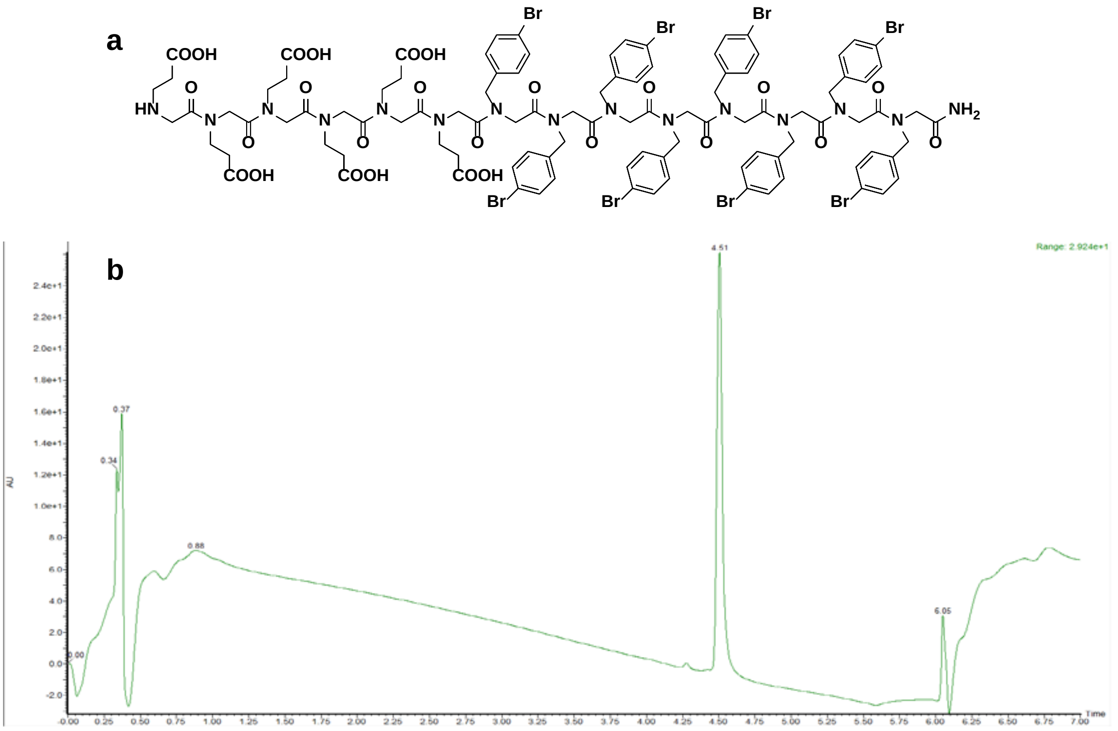

Supplementary Figure 10. Structure of Nce_6Nbpm_8 (**APO10**) (**a**) and UPLC spectrum of APO10 with the gradient of 5~95% CH_3CN in H_2O (**b**).

APO11 [Nce_6Npm_6]: 1673.8 (Molecular Weight), 1674.91 (Found:[M+H]$^+$), 838.70 (Found:[M/2+H]$^+$).

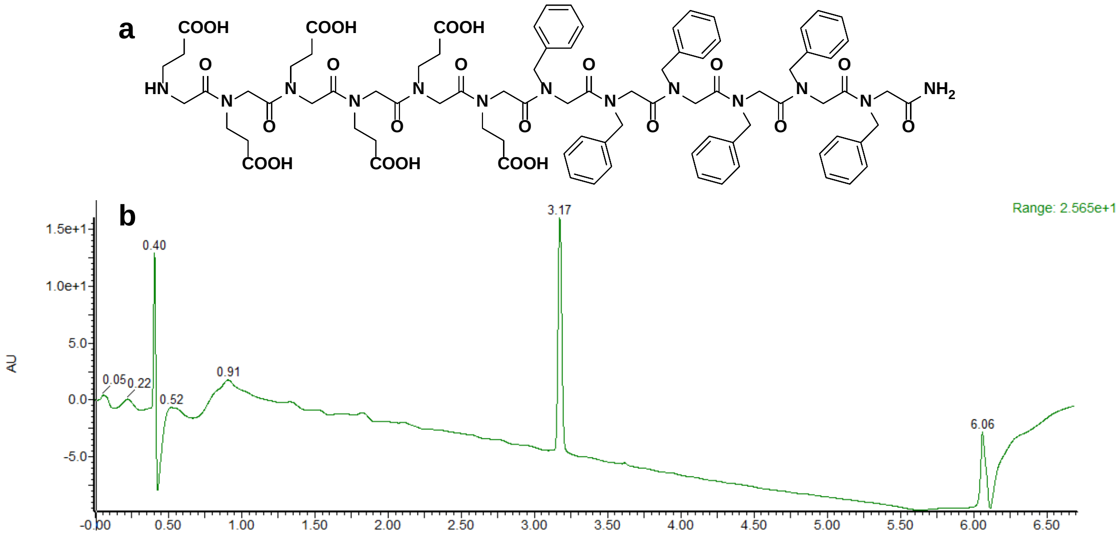

Supplementary Figure 11. Structure of Nce_6Npm_6 (**APO11**) (**a**) and UPLC spectrum of APO11 with the gradient of 5~95% CH_3CN in H_2O (**b**).

Rb-APO2 [Rhodamine B modified Nce_6Nbpm_6]: 2574.68 (Molecular Weight), 2575.36 (Found:$[M+H]^+$) and 1287.54 (Found:$[M/2]^+$)

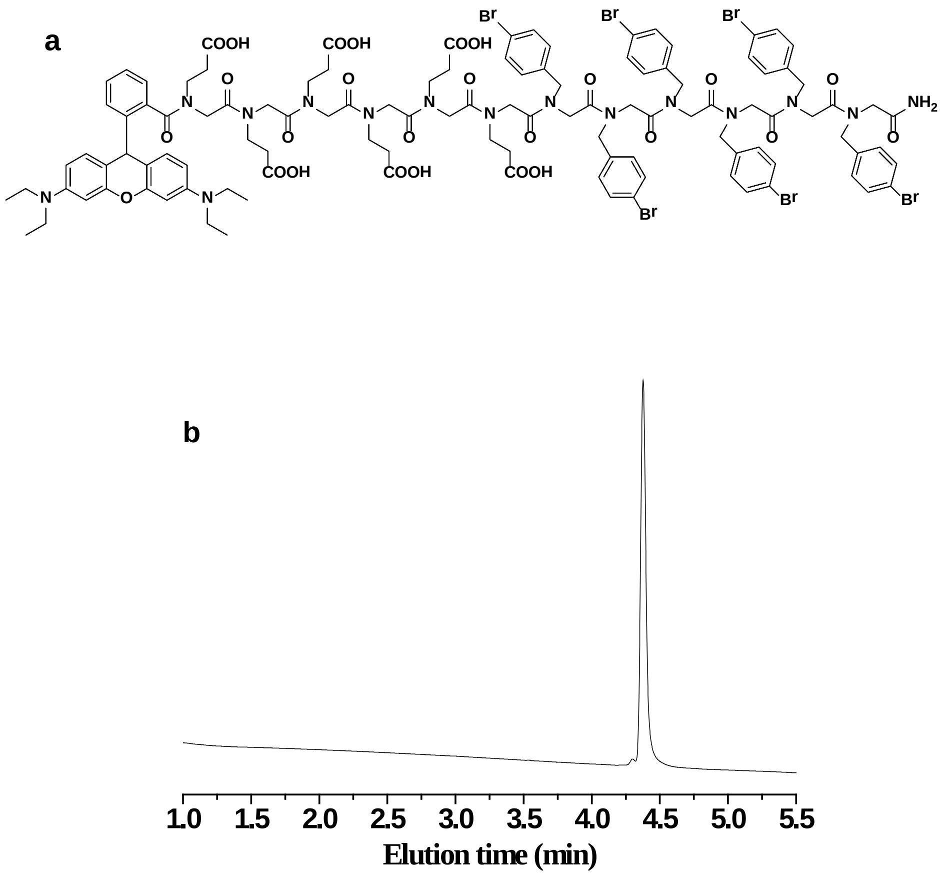

Supplementary Figure 12. Structure of Rhodamine B modified Nce_6Nbpm_6 (**Rb-APO2**) (**a**) and UPLC spectrum of Rb-APO2 with the gradient of 5~95% CH_3CN in H_2O (**b**).

CE-APO2 [15-crown-5 modified Nce$_6$Nbpm$_6$]: 2471.47 (Molecular Weight), 2472.17 (Found:[M+H]$^+$) and 1236.38 (Found:[M/2+H]$^+$)

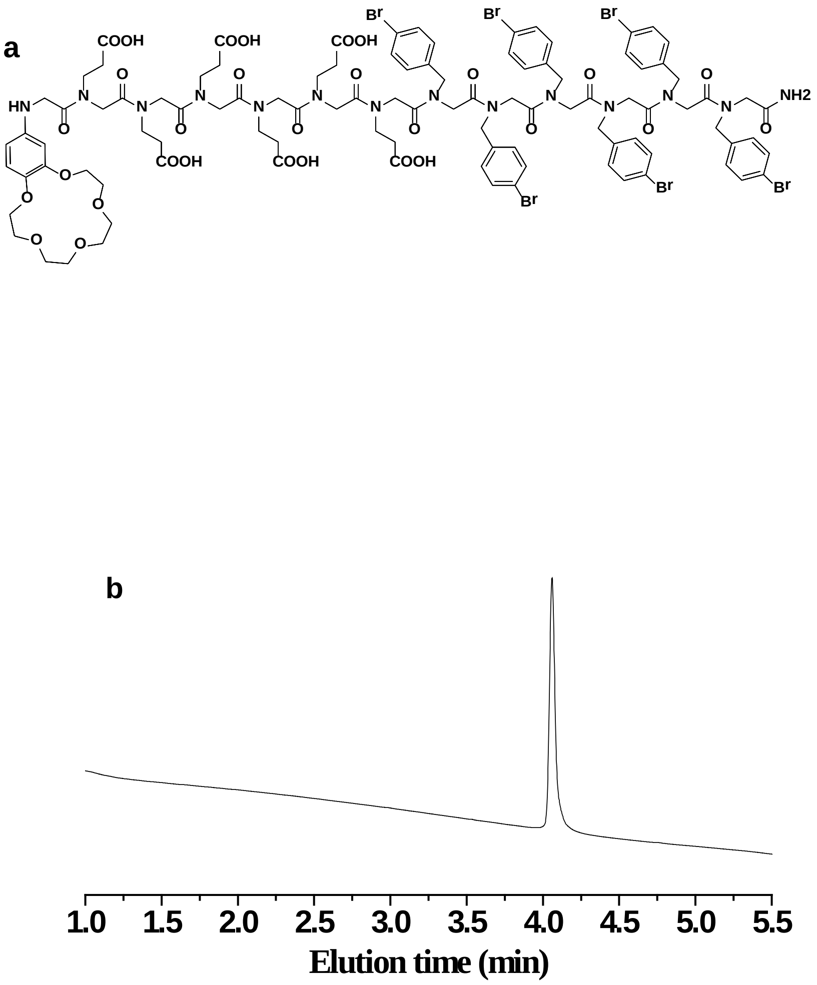

Supplementary Figure 13. Structure of 15-crown-5 modified Nce$_6$Nbpm$_6$ (**CE-APO2**) (**a**) and UPLC spectrum of CE-APO2 with the gradient of 5~95% CH_3CN in H_2O (**b**).

DOP-APO2 [Dopamine modified Nce_6Nbpm_6]: 2341.43 (Molecular Weight), 2341.35 (Found:$[M]^+$) and 1171.27 (Found:$[M/2+H]^+$)

a

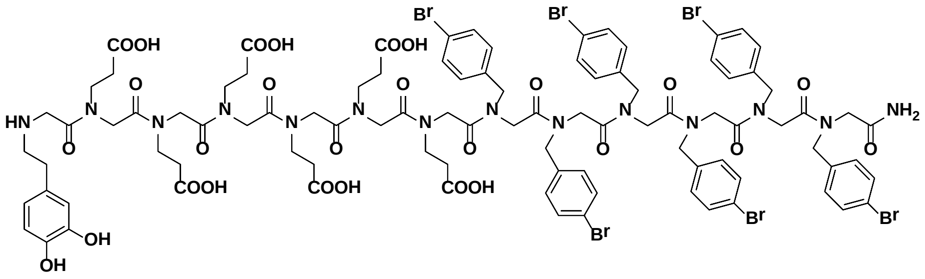

b

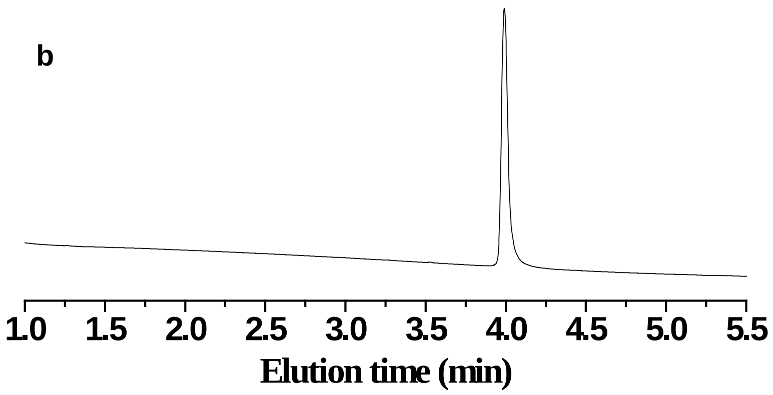

Supplementary Figure 14. Structure of dopamine modified Nce_6Nbpm_6 (**DOP-APO2**) (**a**) and UPLC spectrum of DOP-APO2 with the gradient of 5~95% CH_3CN in H_2O (**b**).

SSYA-APO2 [SSYA modified Nce_6Nbpm_6]: 2556.54 (Molecular Weight), 1279.73 (Found:$[M/2+H]^+$)

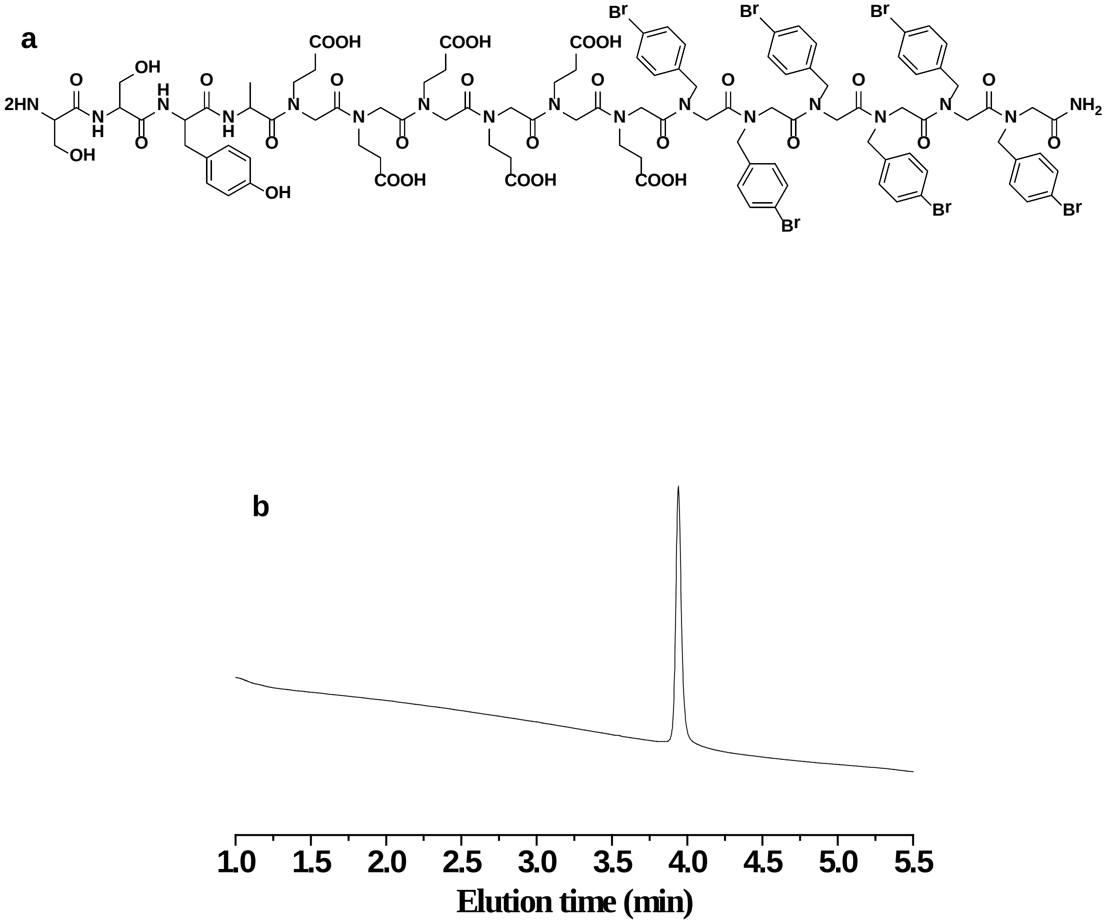

Supplementary Figure 15. Structure of SSYA modified Nce_6Nbpm_6 (**SSYA-APO2**) (**a**) and UPLC spectrum of SSYA-APO2 with the gradient of 5~95% CH_3CN in H_2O (**b**).

FFG-APO2 [FFG modified Nce_6Nbpm_6]: 2499.57 (Molecular Weight), 2500.67 (Found:$[M+H]^+$) and 1250.18 (Found:$[M/2+H]^+$)

a

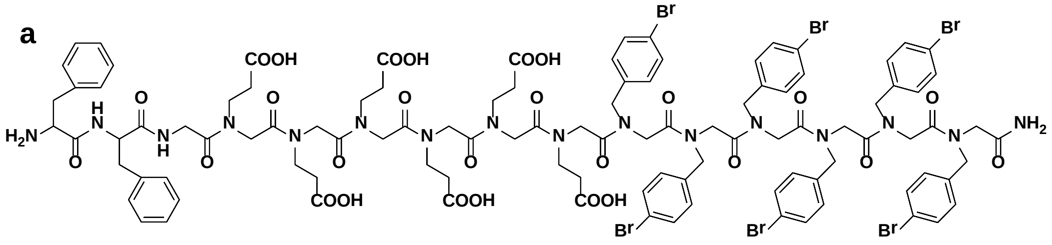

b

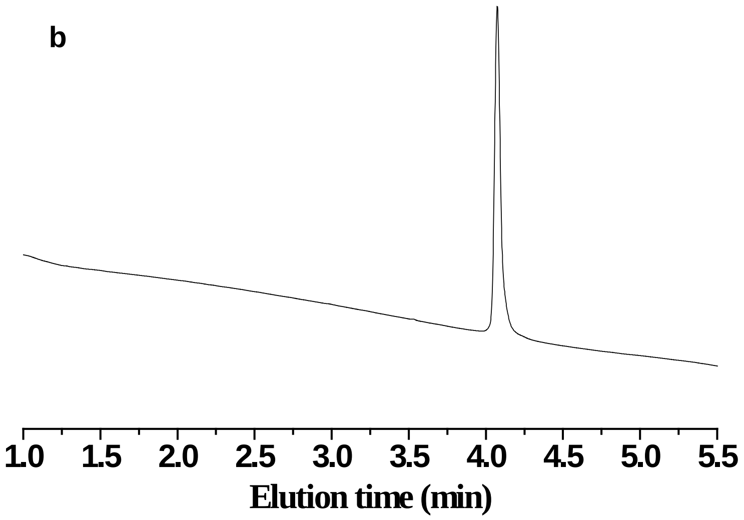

Supplementary Figure 16. Structure of FFG modified Nce_6Nbpm_6 (**FFG-APO2**) (**a**) and UPLC spectrum of FFG-APO2 with the gradient of 5~95% CH_3CN in H_2O (**b**).

CD-APO2 [Cyclodextrin modified Nce_6Nbpm_6]: 3322.18 (Molecular Weight), 1662.39 (Found:$[M/2+H]^+$)

a

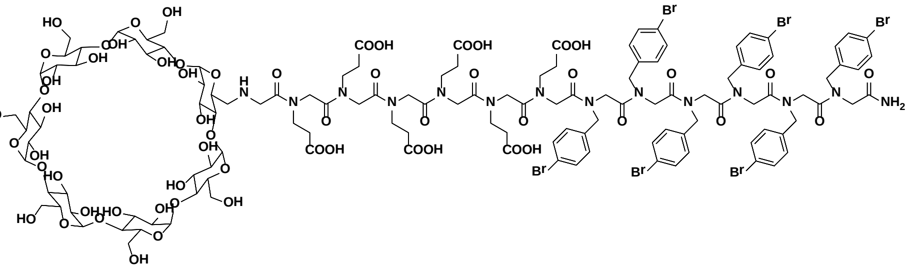

b

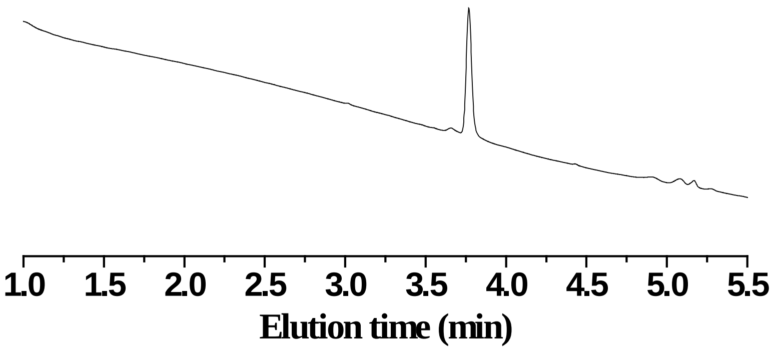

Supplementary Figure 17. Structure of cyclodextrin modified Nce_6Nbpm_6 (**CD-APO2**) (**a**) and UPLC spectrum of CD-APO2 with the gradient of 5~95% CH_3CN in H_2O (**b**).

RGDG-APO2 [RGD-modified Nce$_6$Nbpm$_6$]: 2532.55 (Molecular Weight), 2532.50 (Found:$[M]^+$), 1266.46 (Found:$[M/2]^+$)

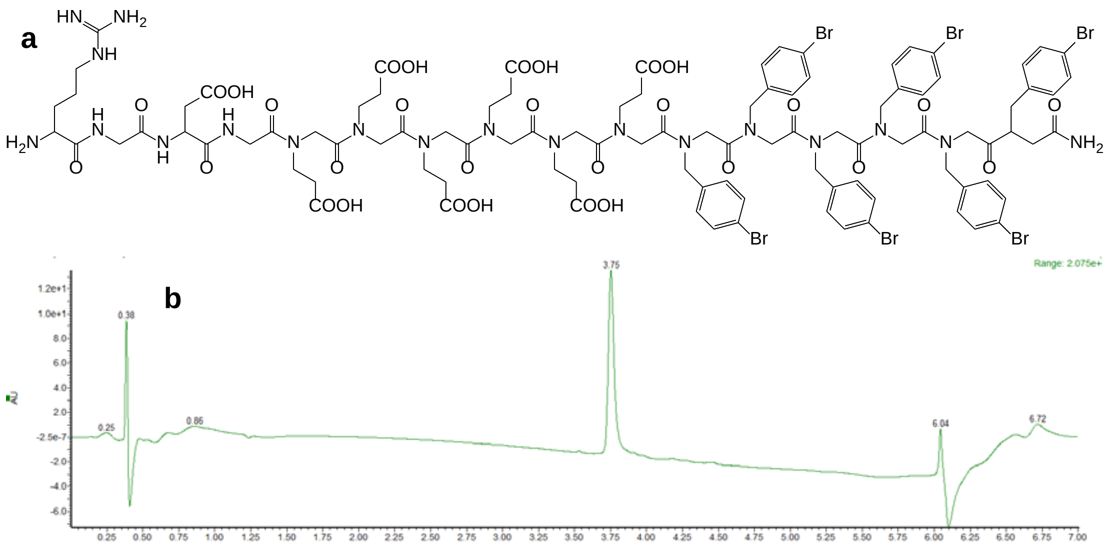

Supplementary Figure 18. Structure of RGD-modified Nce$_6$Nbpm$_6$ (**RGDG-APO2**) (**a**) and UPLC spectrum of RGDG-APO2 with the gradient of 5~95% CH_3CN in H_2O (**b**).

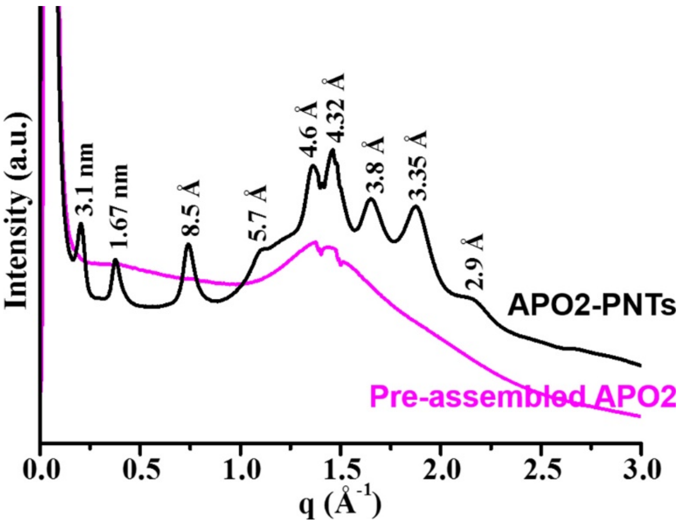

Supplementary Figure 19. Powder X-ray diffraction (XRD) data of pre-assembled APOs and assembled APO2 peptoid nanotubes (PNTs); these data show that pre-assembled APO2 samples are amorphous (In pink) before they crystallize into highly-ordered APO2-PNTs (In black).

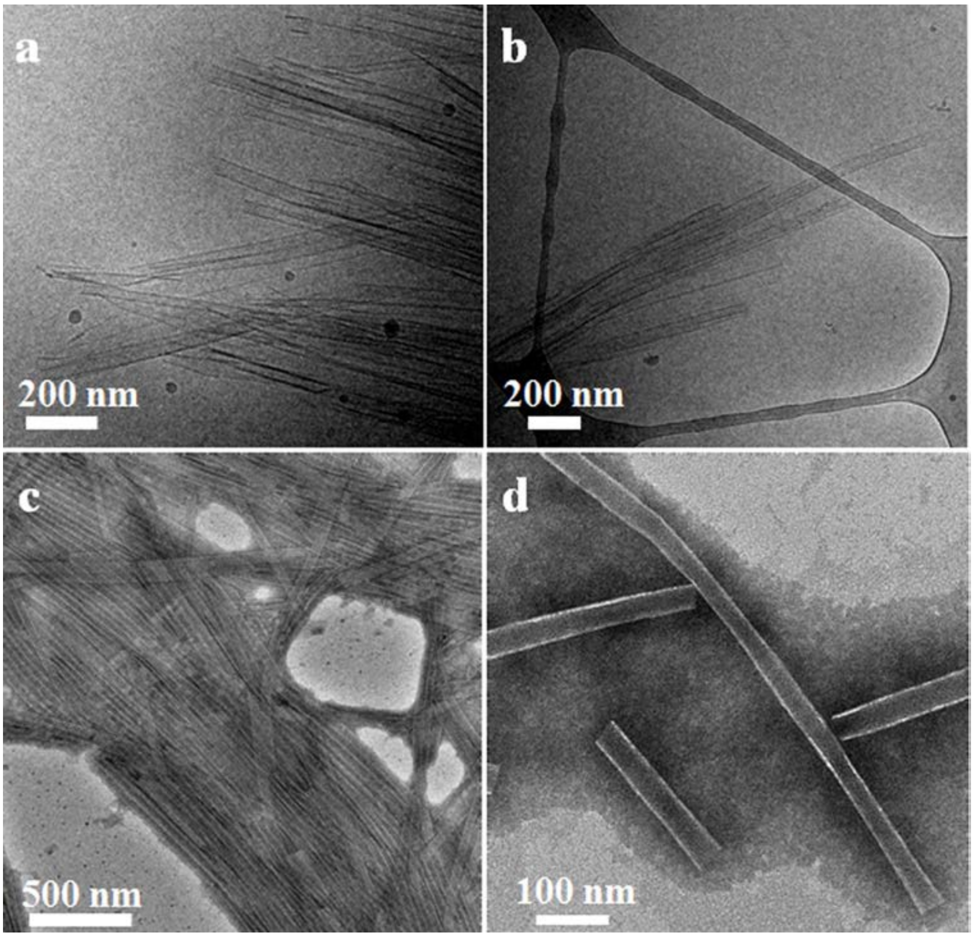

Supplementary Figure 20. (**a** and **b**) Cryo-TEM images of APO2-PNTs without sonication. (**c** and **d**) TEM images of APO2-PNTs. (**c**) TEM image of APO2-PNTs without sonication; (**d**) TEM image of APO2-PNTs with sonication.

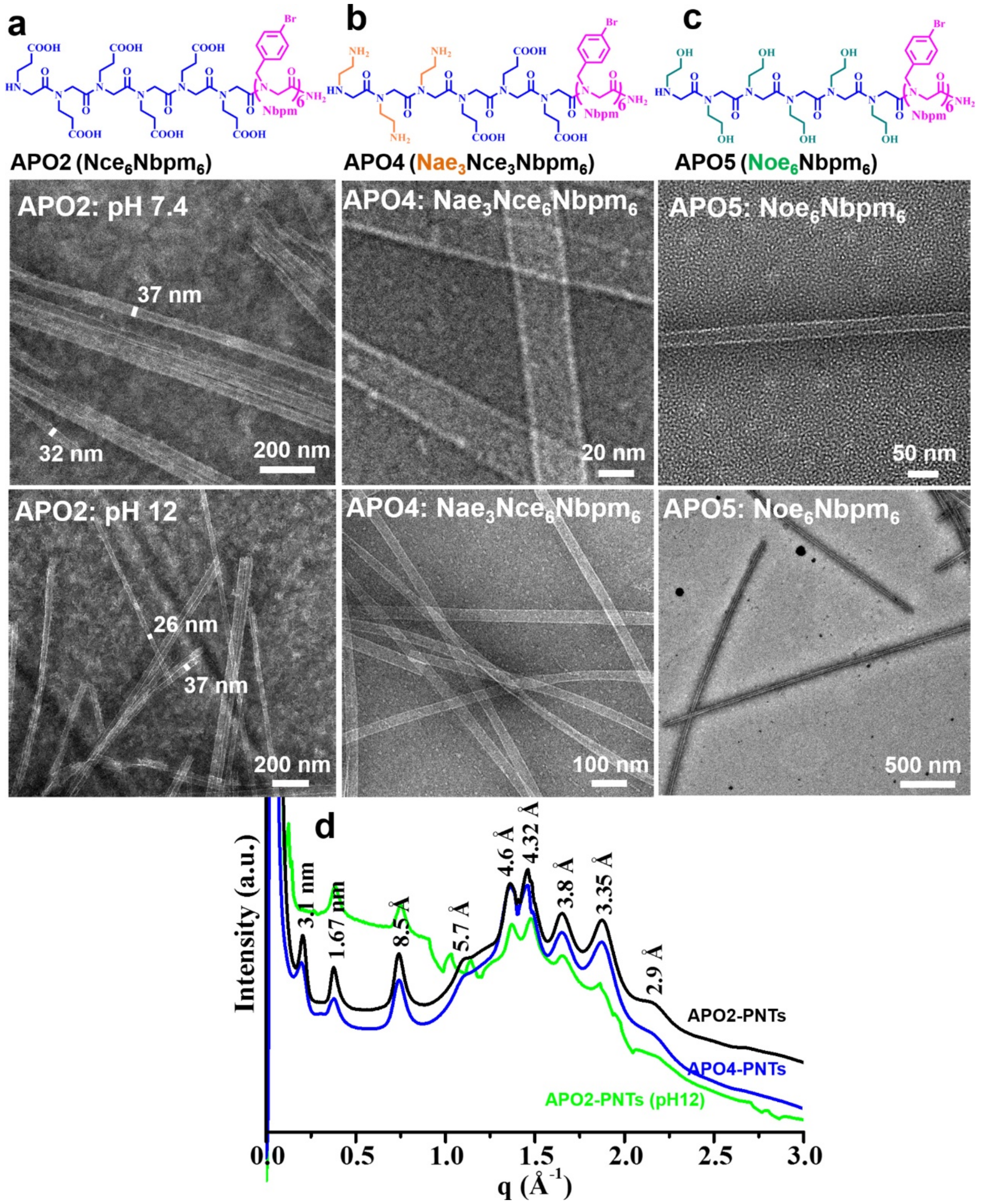

Supplementary Figure 21 | The ordering of hydrophobic domains is the key to forming tubular structures. (**a**) Structure of APO2 (Nce_6Nbpm_6) and TEM images of APO2-PNTs crystallized at pH 7.4 and pH 12. (**b**) Structure of APO4 ($Nae_3Nce_3Nbpm_6$) and TEM images of APO4-PNTs; while six Nbpm group were remained, changing three Nce groups to positively charged three Nae groups didn't disrupt the tubular structure formation. (**c**) Structure of APO5 (Noe_6Nbpm_6) [Noe = N-(2-hydroxylethyl)glycine] and TEM images of APO5-PNTs; TEM data show that APO5 assembled into tubes even with six polar Noe residues as the hydrophilic domain. (**d**) XRD data of regular APO2-PNTs, APO4-PNTs and PNTs assembled from APO2 at pH12 show that all of these tubes exhibit similar structures.

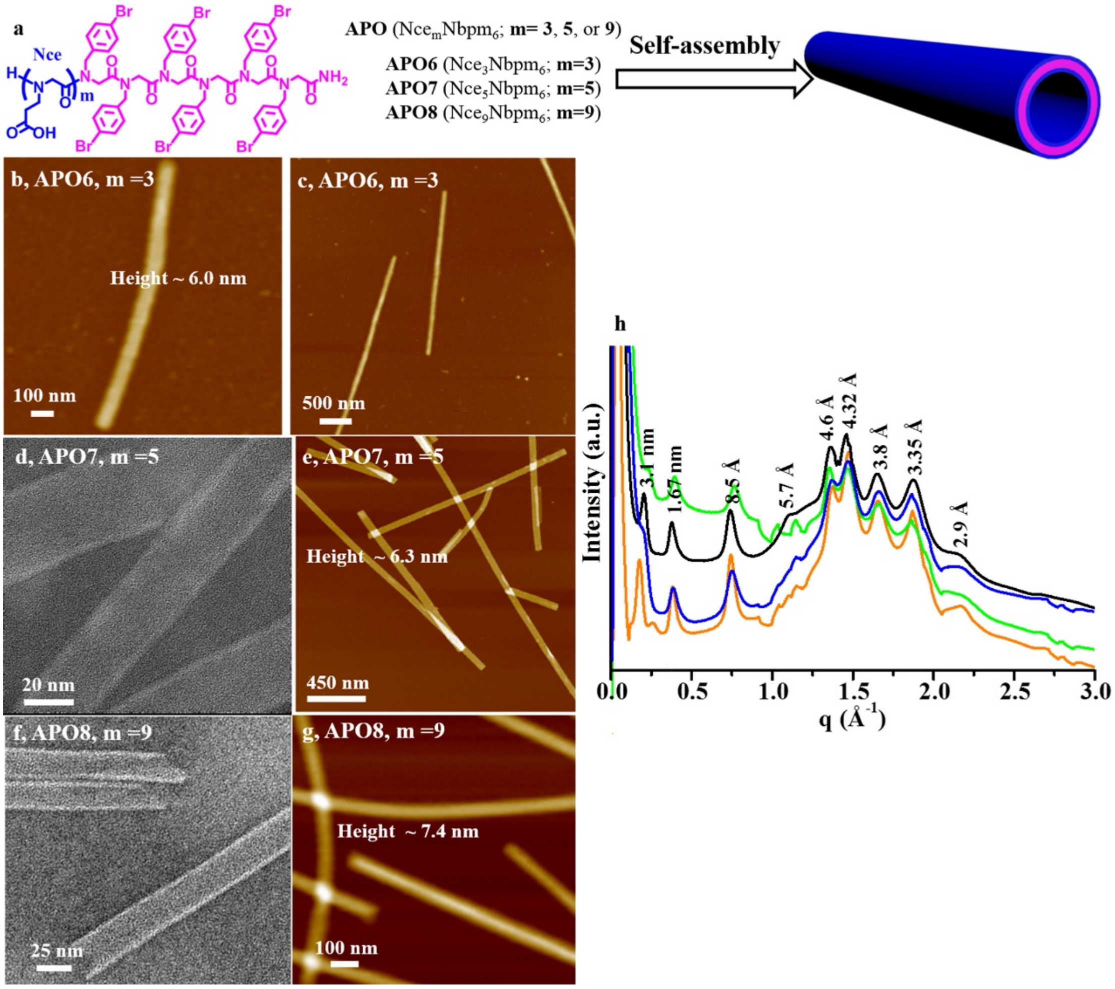

Supplementary Figure 22. (**a**) Structures of APOs (Nce$_m$Nbpm$_6$: APO6, m = 3; APO7, m = 5; APO8, m = 9) with variable number of Nce groups, and the scheme showing the assembly of APOs into SW-PNTs. (**b** and **c**) AFM images of APO6-PNTs. (**d** and **e**) TEM image (d) and AFM height image (**e**) of APO7-PNTs. (**f** and **g**) TEM image (**f**) and AFM height image (**g**) of APO8-PNTs. (**h**) X-ray diffraction (XRD) data of PNTs assembled from APO6 (Green), APO7 (Blue), or APO8 (Orange); all of these PNTs show similar XRD peaks to those of APO2-PNTs (Black), indicating they all have similar structures.

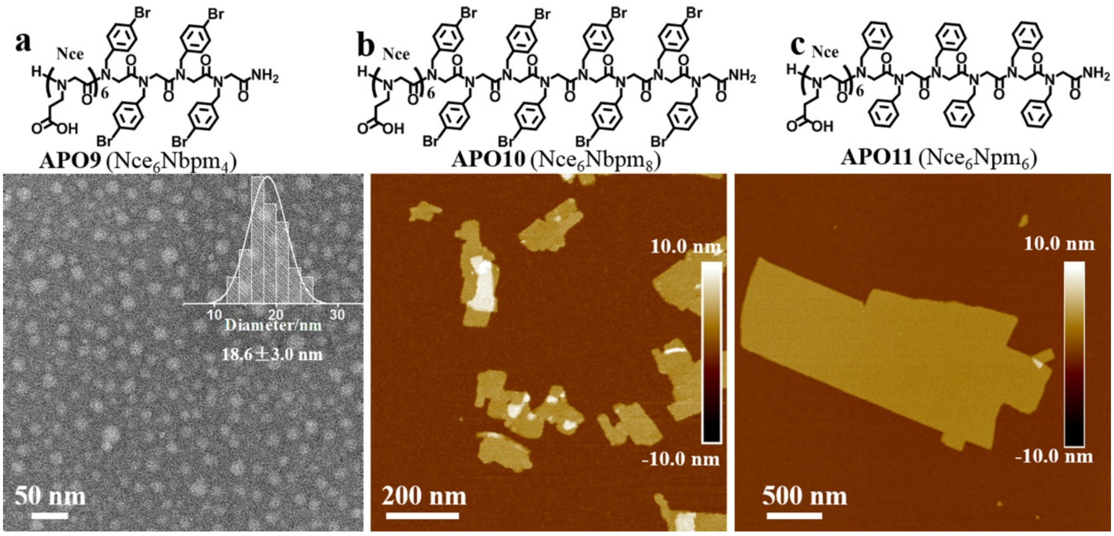

Supplementary Figure 23. (**a**) Structure of APO9 (Nce_6Nbpm_4) and TEM image of micelle-like particles assembled from APO9; the insert is the statistical size distribution showing the micelle diameter is 18.6 ± 3.0 nm. (**b**) Structure of APO10 (Nce_6Nbpm_8) and AFM height image of nanosheets (AFM height of ~4.0 nm) assembled from APO10. (**c**) Structure of APO11 (Nce_6Npm_6) and AFM height image of nanosheets (AFM height of ~3.6 nm) assembled from APO11. The assembly of these three peptoids into non-tubular structures confirmed the importance of hydrophobic domains in the formation of tubular structure.

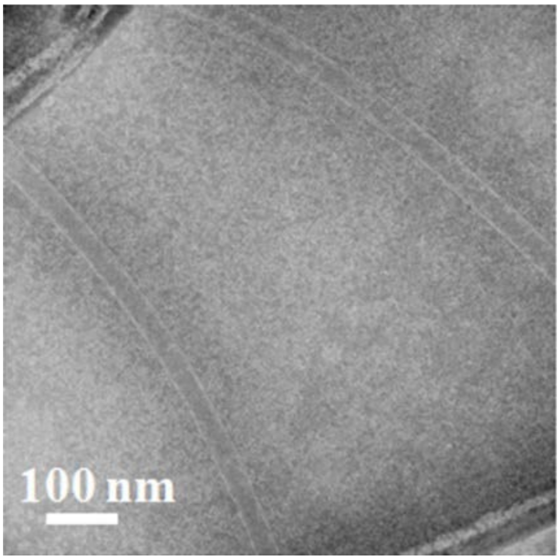

Supplementary Figure 24. TEM image of PNTs that were formed after 5.0 mM CH_3CN &H_2O (1:1) solution of APO2 was left for only about 0.5 hours.

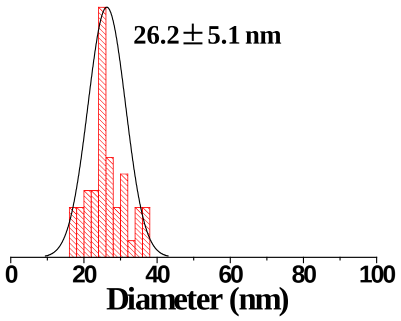

Supplementary Figure 25. Statistical size distribution of nanospheres formed at the beginning of PNT assembly process. The number above histogram is the average spherical diameter; 50 particles were analyzed for each size distribution.

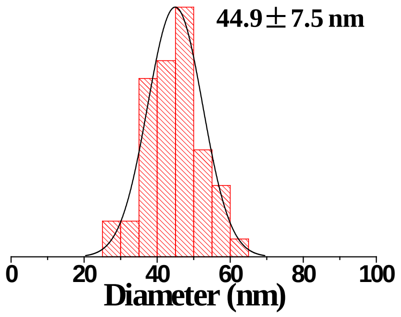

Supplementary Figure 26. Statistical size distribution of nanospheres formed at the half hour of PNT assembly process. The number above histogram is the average spherical diameter; 50 particles were analyzed for each size distribution.

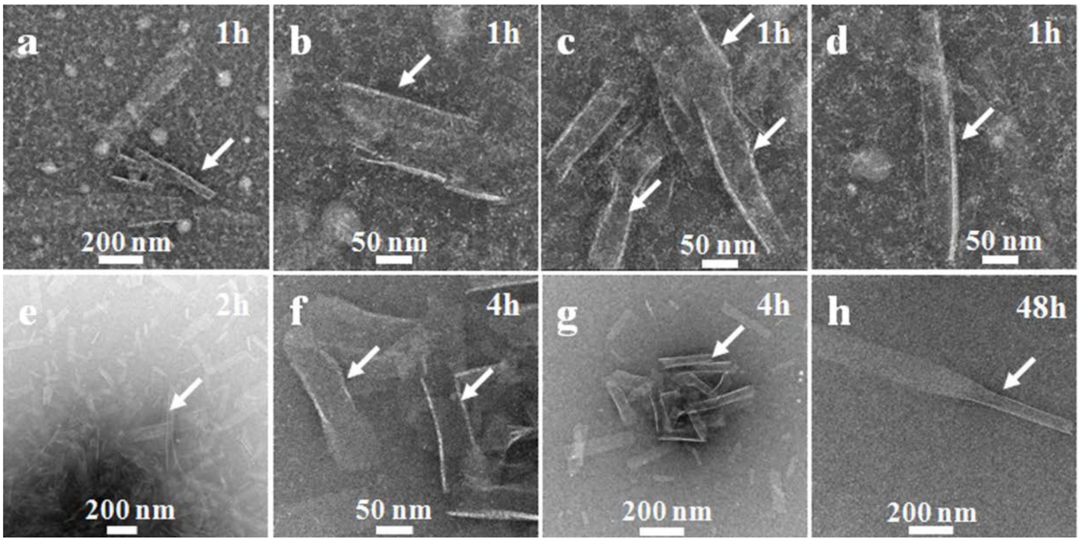

Supplementary Figure 27. Negatively-stained TEM images showing the ribbon-like objects began to roll up. The process was observed during the assembly time from 1 hour to 48 hours.

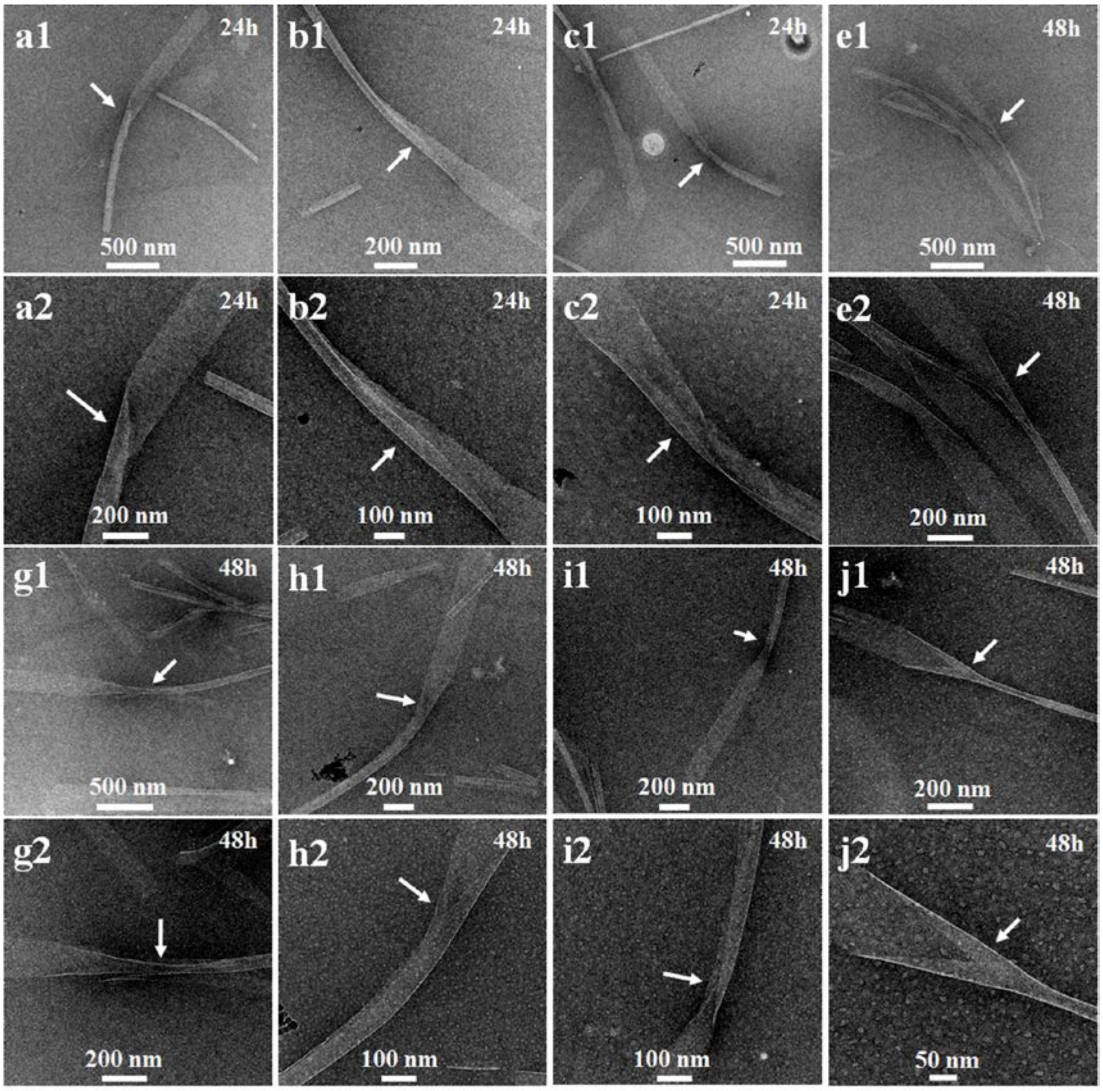

Supplementary Figure 28. Negatively-stained TEM images showing that the ribbon-like objects began to fold. The process could be easy observed after 1 day. Figs. a1 - j1 (low-magnification TEM images) showing the whole structures of intermediates of PNTs, and about half PNTs and half nanosheets were observed. Figures a2 - j2 are high-magnification TEM images showing the joint domains between PNTs and nanosheets, in which the folding and closing up of nanosheets can be observed.

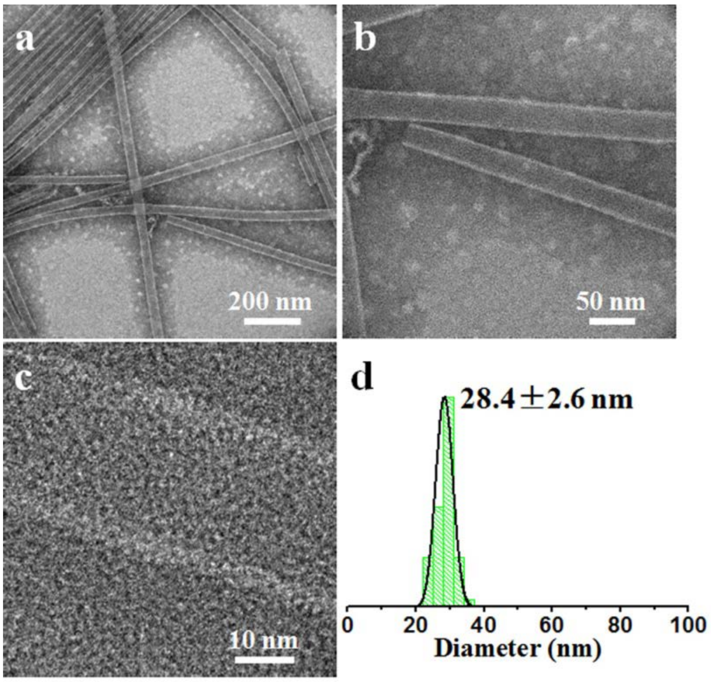

Supplementary Figure 29. Characterizations of APO1-PNTs. (**a - c**) TEM images; (**d**) Statistical size distribution. The number above histogram is the average tubular diameter; 50 nanotubes were analyzed for each size distribution.

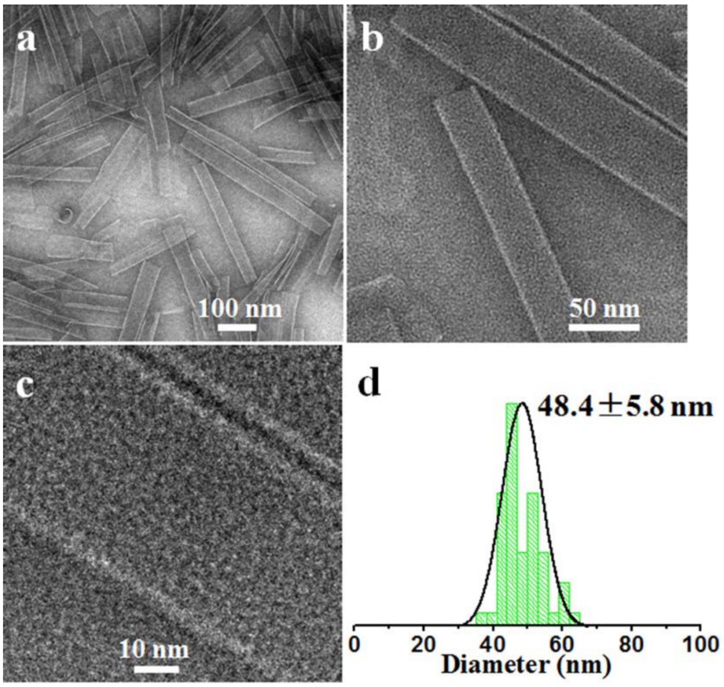

Supplementary Figure 30 | Characterizations of APO3-PNTs. (**a - c**) TEM images; (**d**) Statistical size distribution. The number above histogram is the average tubular diameter; 50 nanotubes were analyzed for each size distribution.

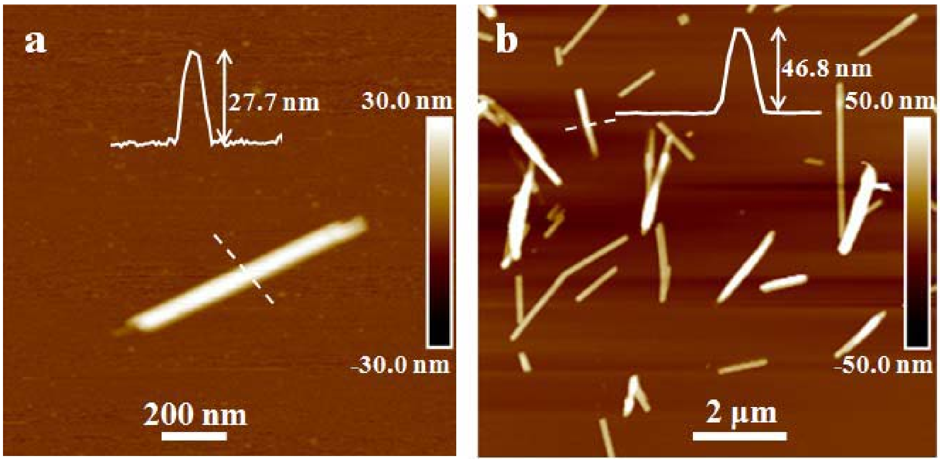

Supplementary Figure 31. *In-situ* AFM height images of APO1-PNTs (**a**) and APO3-PNTs (**b**). The insets were the height curves showing the PNT heights in the liquid environments.

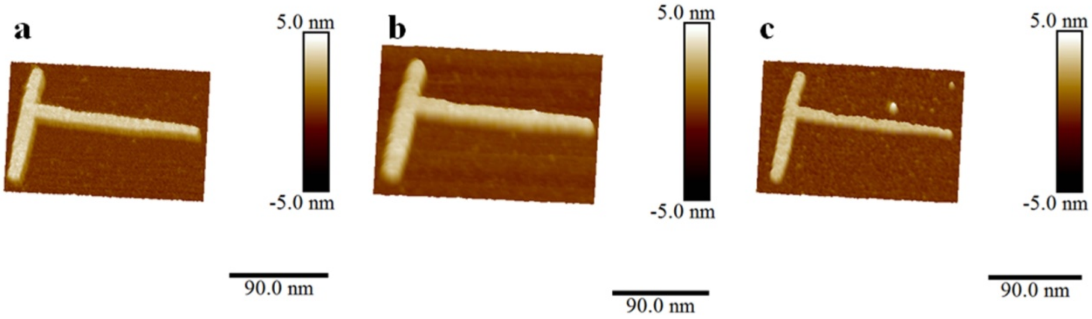

Supplementary Figure 32. (**a** - **c**) *In-situ* AFM height images of APO2-PNTs after being incubated in the water. (**a**) 0 hour; (**b**) for 1 hour; (**c**) for 2 hours.

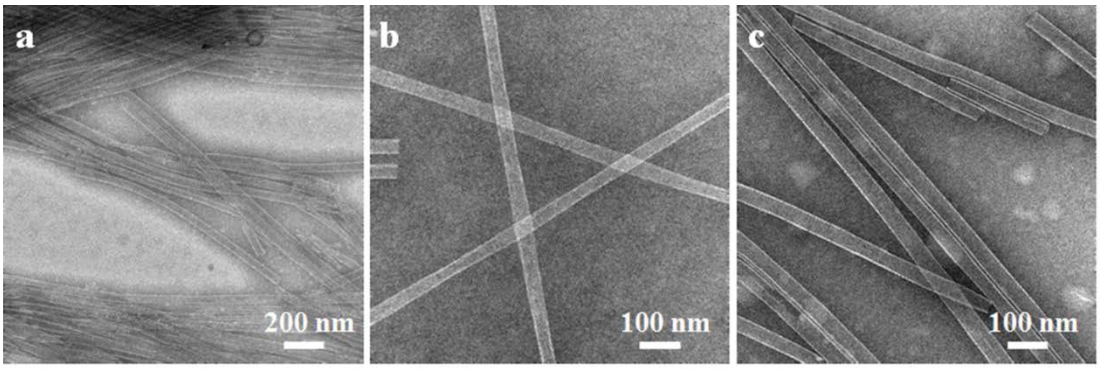

Supplementary Figure 33 | Stability test of APO2-PNTs. (**a**) TEM images of APO2-PNTs after being incubated in H_2O/CH_3CN (v/v=1:1) mixture for 3 hours; (**b**) TEM images of APO2-PNTs after being incubated in 1X PBS buffer for 3 hours; (**c**) TEM images of APO2-PNTs after being incubated in 60 ^{o}C for 3 hours.

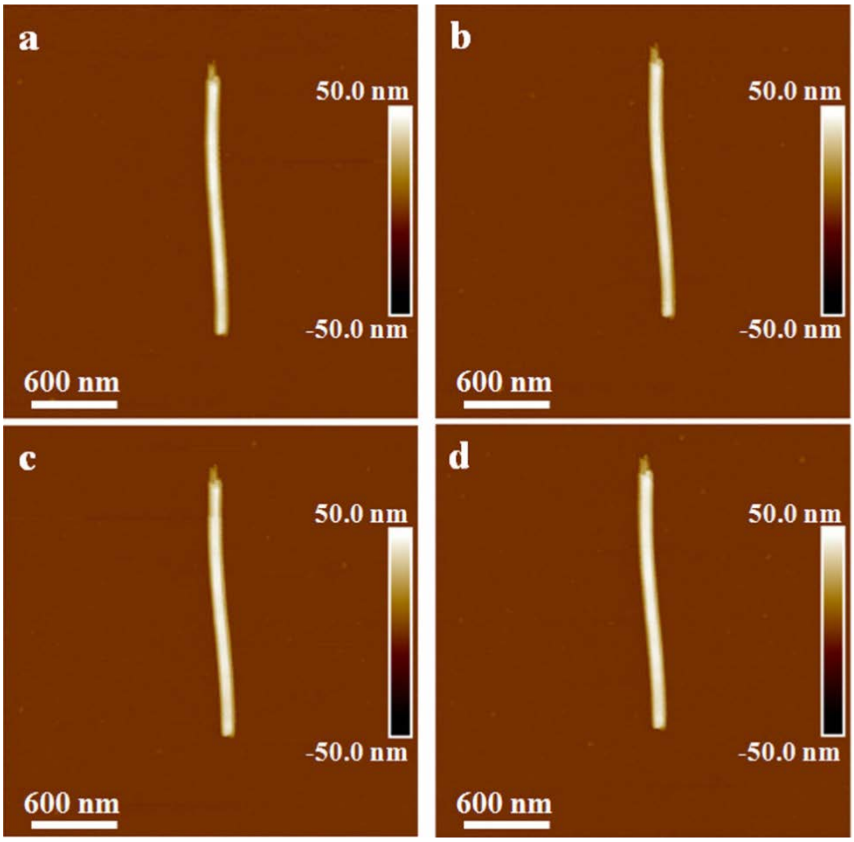

Supplementary Figure 34. *In-situ* AFM height images of APO2-PNTs in NaCl aqueous solution. (**a**) In pure water; (**b**) In 0.25 M NaCl aqueous solution; (**c**) In 0.5 M NaCl aqueous solution; and (**d**) in 1.0 M NaCl aqueous solution.

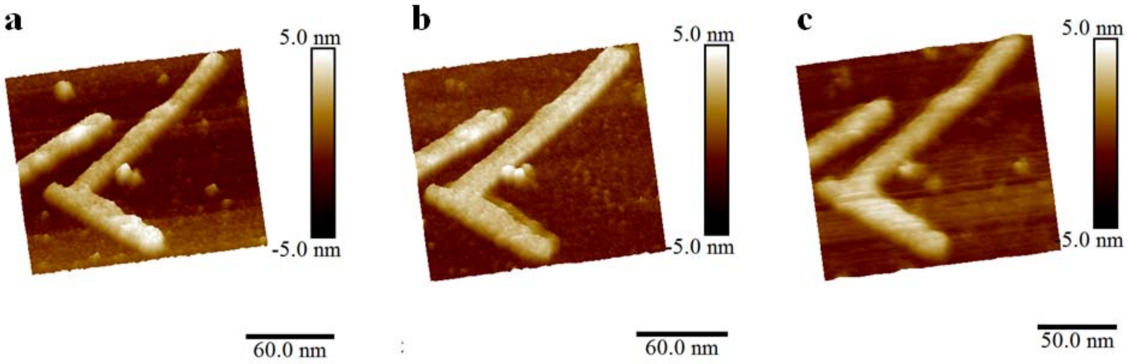

Supplementary Figure 35. (**a-c**) *In-situ* AFM height images of APO2-PNTs after being incubated in the pure CH_3CN, for (**a**) 0 hour; (**b**) 1 hour; and (**c**) 2 hours.

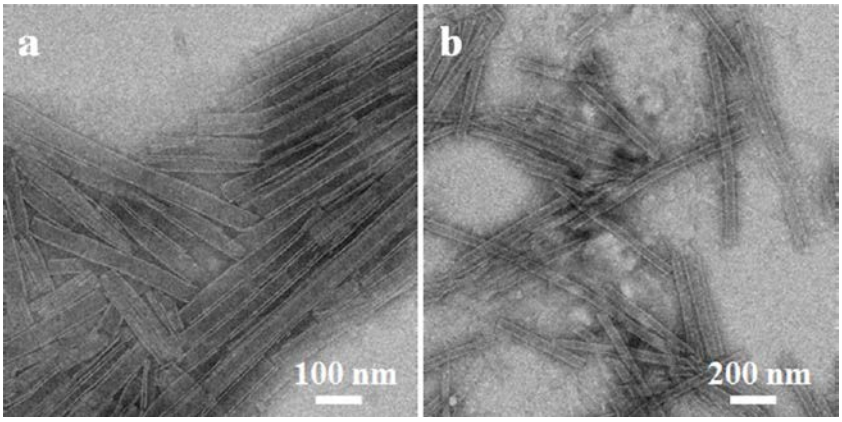

Supplementary Figure 36 | Stability test of APO2-PNTs. (**a**) TEM images of APO2-PNTs after being incubated in ethanol for 3 hours; (**b**) TEM images of APO2-PNTs after being incubated acetonitrile for 3 hours.

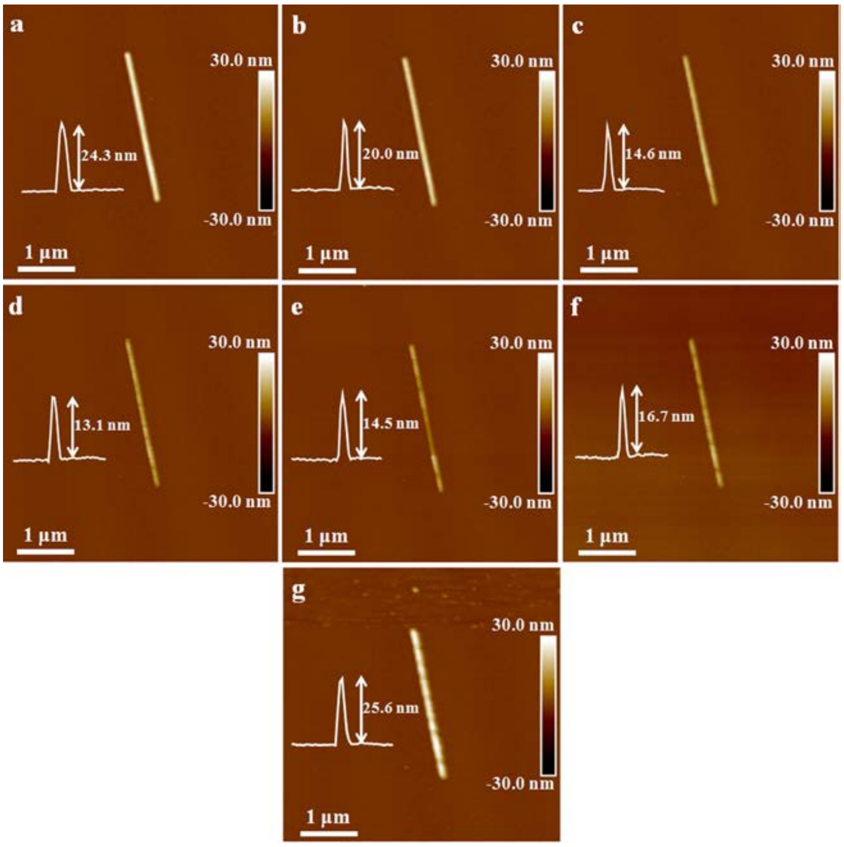

Supplementary Figure 37 | *In-situ* AFM height images of APO2-PNTs in aqueous solution at different pH. (**a**) pH 8.0, (**b**) pH 7.0, (**c**) pH 5.4, (**d**) pH 3.6, (**e**) pH 5.4, (**f**) pH 7.0, (**g**) pH 8.0. NaOH and trifluoroacetic acid (TFA) were employed to adjust solution pH. AFM imaging was performed in fluid cell with PFQNM mode. After AFM imaging, the fluid cell was washed three times by 100 mL of testing solution in the next step. It was revealed that PNT's diameter was reversibly responsive to pH change.

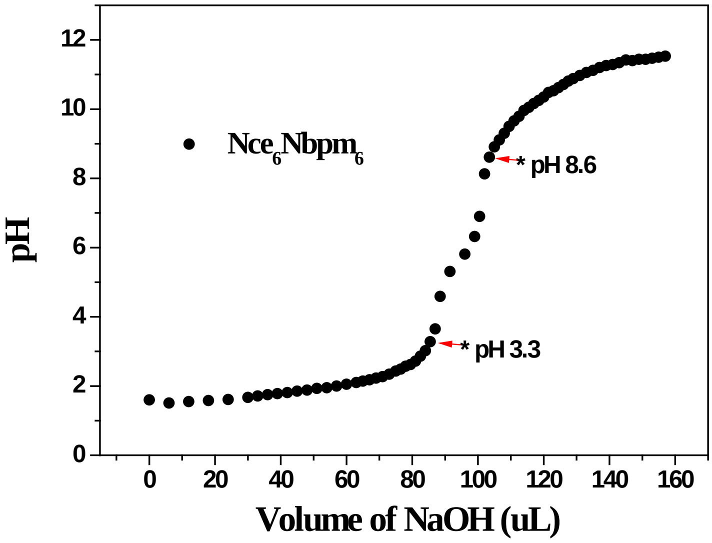

Supplementary Figure 38 | Acid-base titration curve of Nce_6Nbpm_6 (APO2). The titration curve for APO2 was performed on Mettler Toledo™ S220 SevenCompact™ pH/Ion Benchtop Meter. Because APO2 is not soluble in water, for this titration study, 2 μmol of APO2 were dissolved in the mixture of 1.0 mL of H_2O and 15 μL of 2.0 M NaOH (final solution pH is12.49). 125 μL of the resulting clear solution was further diluted with 125 μL of H_2O to obtain the 1.0 mM APO2 aqueous solution (the final solution pH is 12.14). After adding 16 μL of 1.0 M HCl into this 1.0 mM peptoid solution (the final solution pH is 1.60), NaOH aqueous solution (0.1 M) was stepwise added to adjust the pH in the titration process. The point before pH 3.3 is probably where all carboxyl groups of APO2 are protonated. The region from pH 3.3 to pH 8.6 is where the carboxyl groups of APO2 start to become partially and then fully deprotonated.

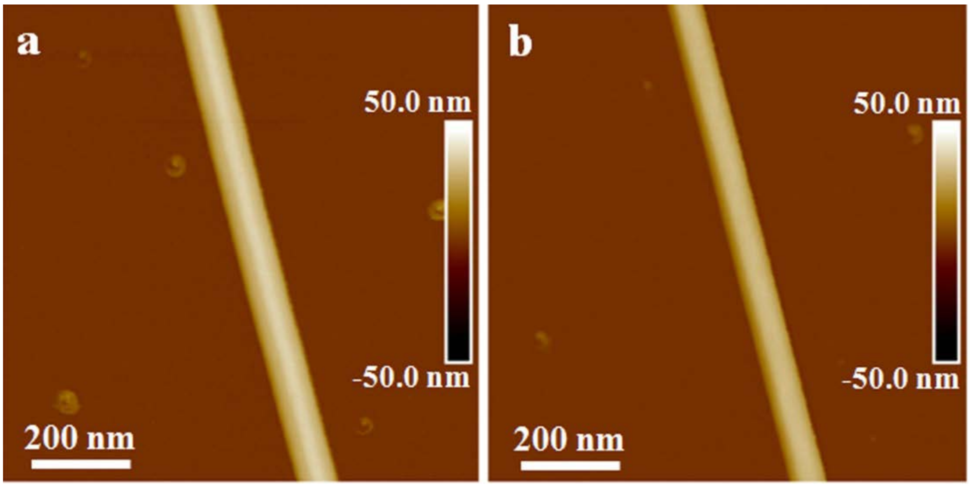

Supplementary Figure 39. AFM height images of one APO3-PNT before (**a**) and after (**b**) scanning in the aqueous solution. This PNT maintains the tubular features in spite of the nanoindentation. Instant recovery of diameter is observed, which indicates that only elastic deformation occurs in the indentation process.

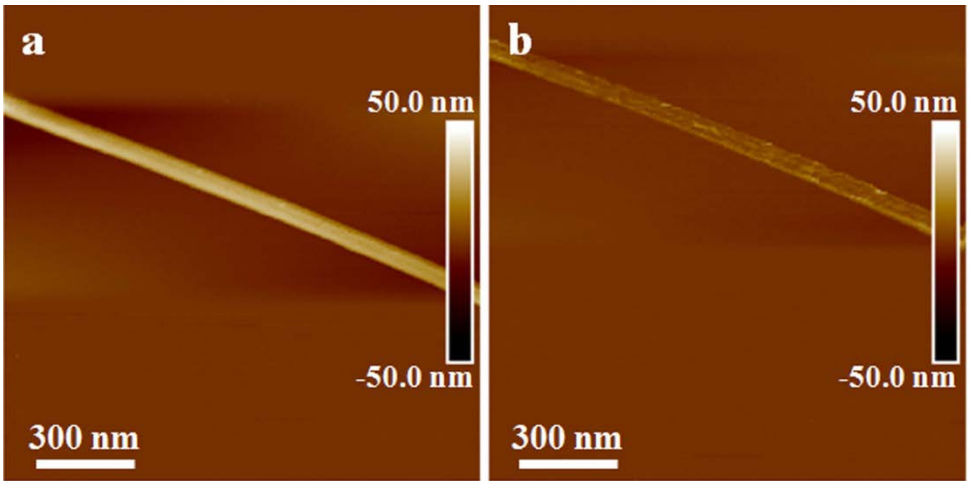

Supplementary Figure 40. AFM height images of one APO1-PNT before (**a**) and after (**b**) scanning in the aqueous solution. This PNT was unstable under this condition and we observed the PNT breakdown. The stronger force tapped on PNT's surface led to the disassembly and dissolution of peptoid molecules.

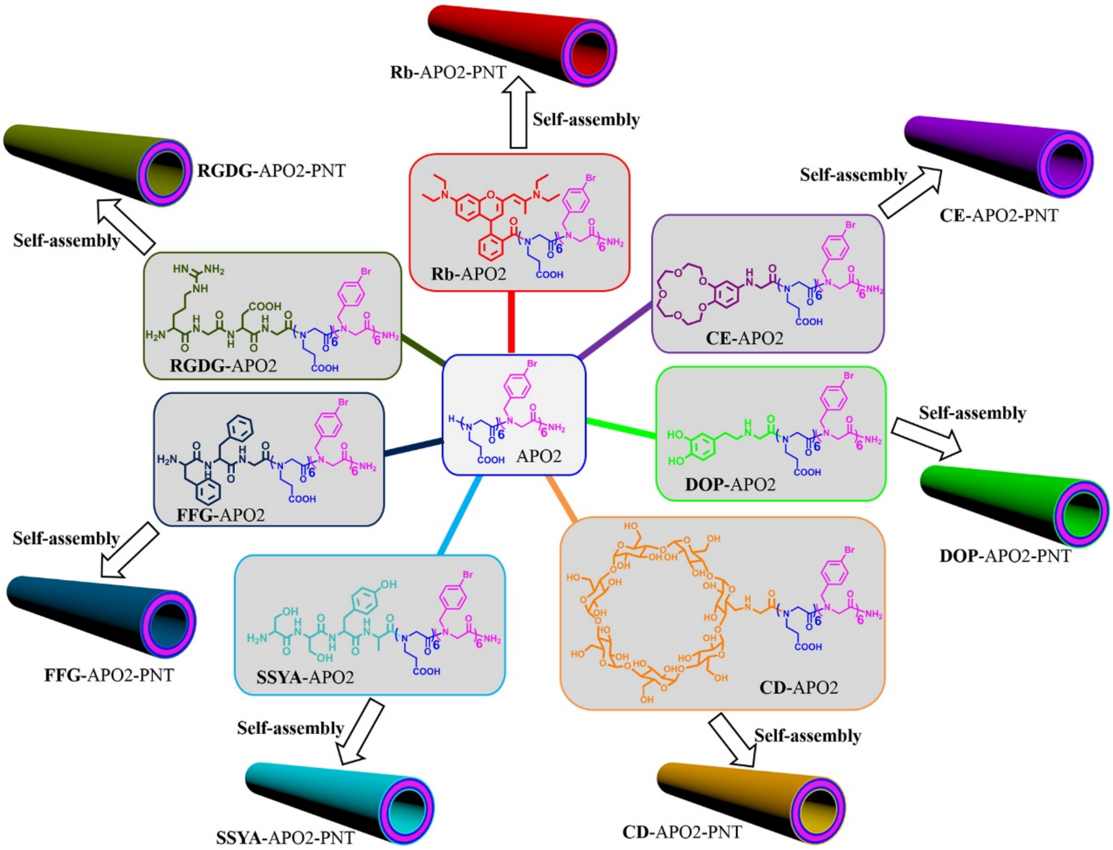

Supplementary Figure 41 | Incorporation of functional groups/molecules into APO2 for assembly of functionalized nanotubes. Structures of APO2 and its seven functionalized APOs, and the scheme showing the assembly of these functionalized APOs into SW-PNTs. The hydrophobic domain of APO2 is highlighted in pink color, the polar domain in blue color, and the functional groups/molecules of these APO2 in the colors indicated by the labels. The latter are located in both exterior and interior surfaces of the nanotubes.

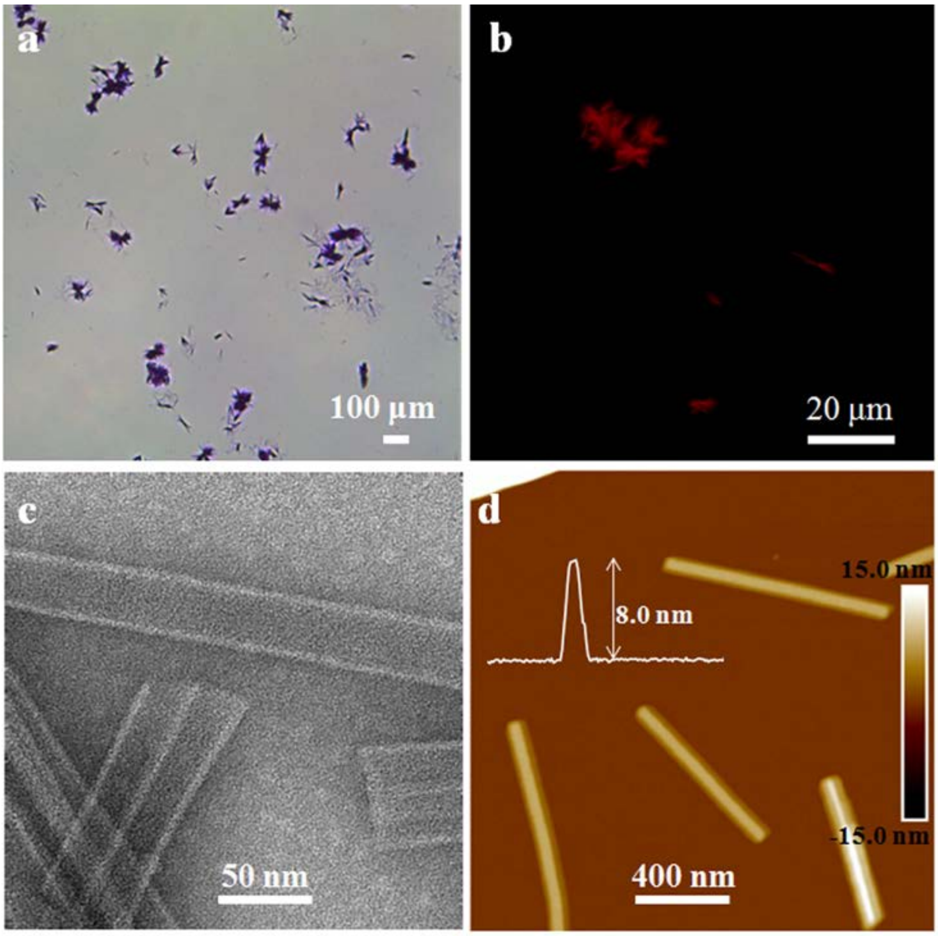

Supplementary Figure 42 | Characterizations of Rb-APO2-PNTs. (**a**) Optical microscopy image of **Rb**-APO2-PNTs; (**b**) Confocal laser scanning microscopy (CLSM) image of **Rb**-APO2-PNTs; these PNTs exhibited red color due to the fluorescence of **Rb** (Rodamine B); (**c**) TEM image of **Rb**-APO2-PNTs; (**d**) AFM height image of **Rb**-APO2-PNTs; the inset is a PNT height curve.

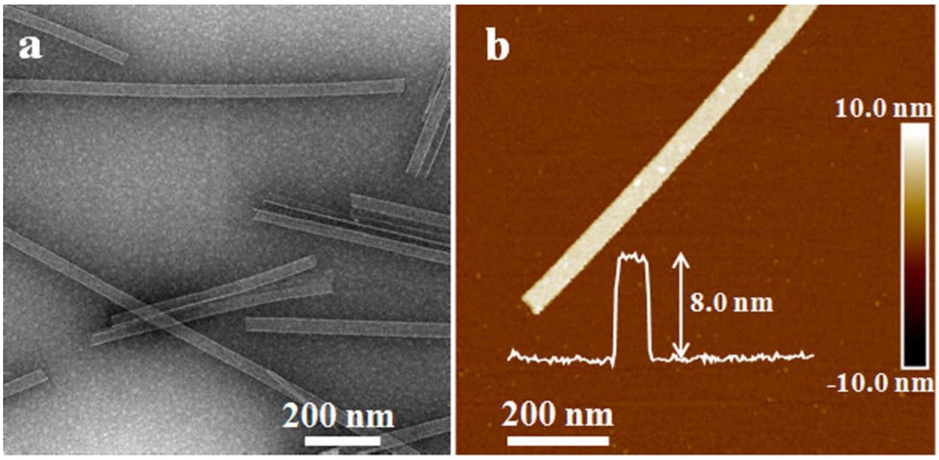

Supplementary Figure 43 | Characterizations of CE-APO2-PNTs. (**a**) TEM image of **CE**-APO2-PNTs; (**b**) AFM height image of **CE**-APO2-PNTs; the inset is a PNT height curve.

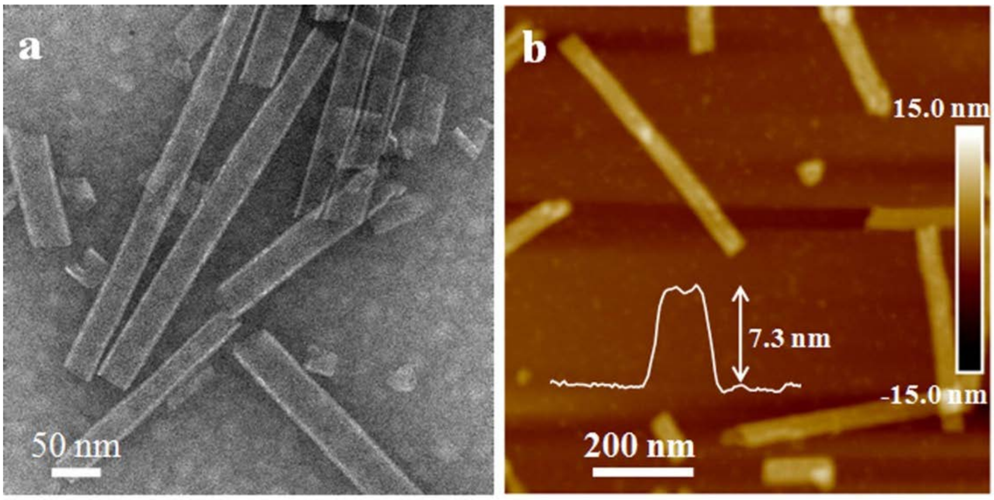

Supplementary Figure 44 | Characterizations of DOP-APO2-PNTs. (**a**) TEM image of **DOP-**APO2-PNTs; (**b**) AFM height image of **DOP-**APO2-PNTs; the inset is a PNT height curve.

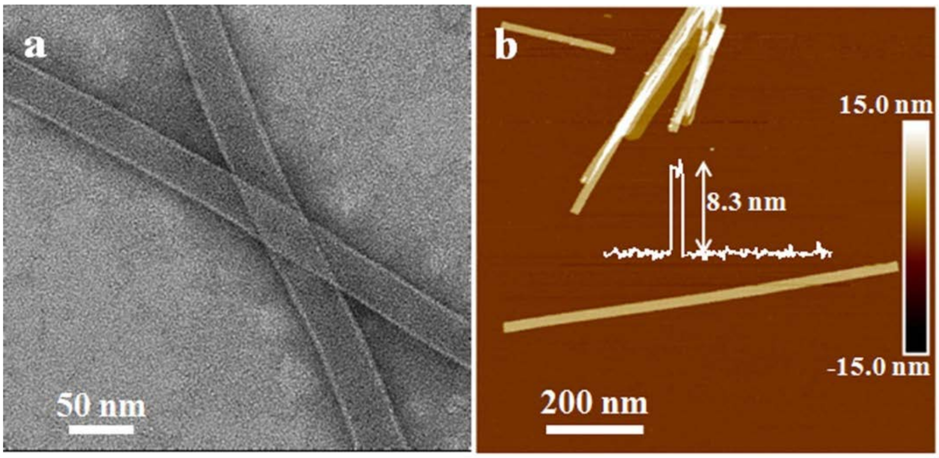

Supplementary Figure 45 | Characterizations of SSYA-APO2-PNTs. (**a**) TEM image of **SSYA-**APO2-PNTs; (**b**) AFM height image of **SSYA-**APO2-PNTs; the inset is a PNT height curve.

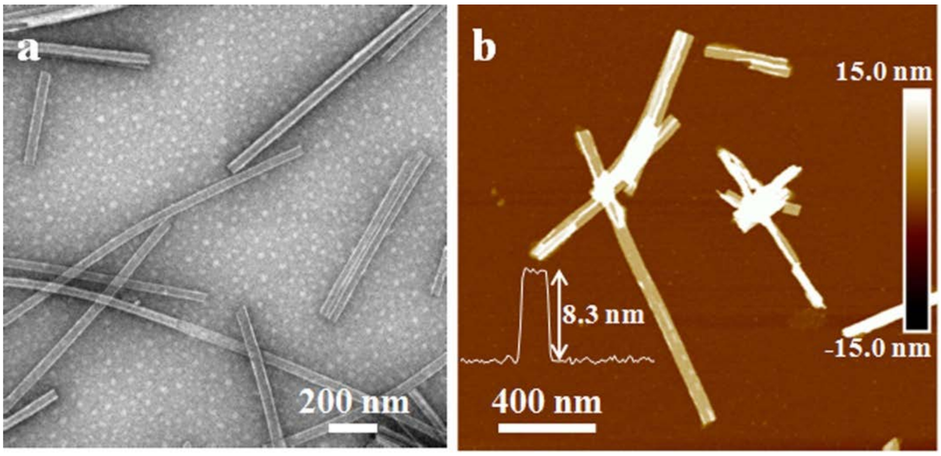

Supplementary Figure 46 | Characterization of FFG-APO2-PNTs. (**a**) TEM image of **FFG-**APO2-PNTs; (**b**) AFM height image of **FFG-**APO2-PNTs; the inset is a PNT height curve.

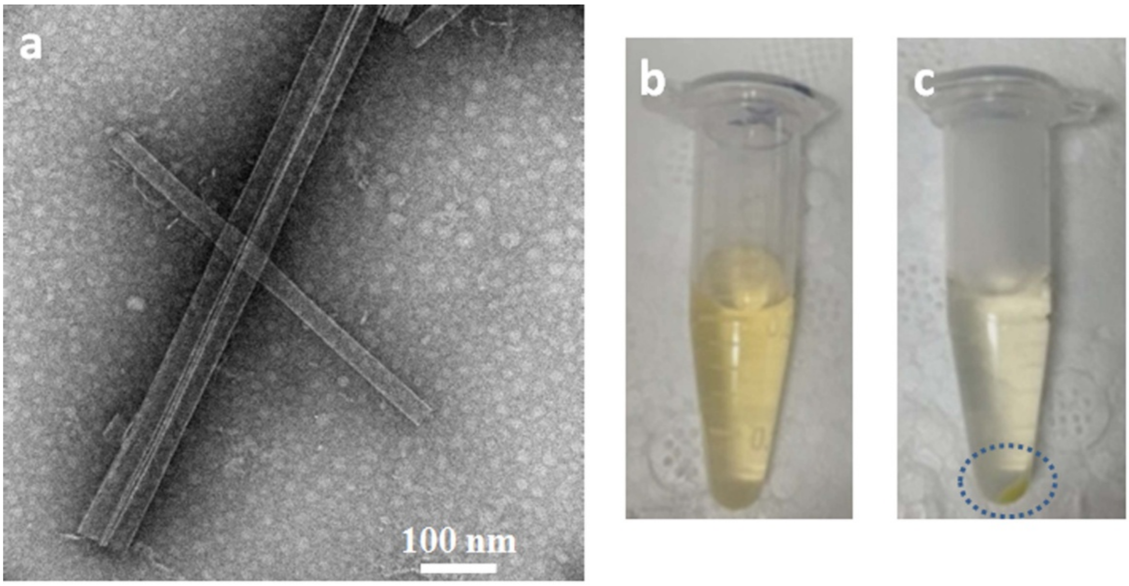

Supplementary Figure 47. (**a**) TEM image of PNTs assembled from cyclodextrin modified Nce_6Nbpm_6 (**CD-APO2**). (**b** and **c**) 4-aminoazobenzene aqueous solution. (**b**) Before incubation with CD-PNTs. (**c**) After incubation with CD-PNTs.

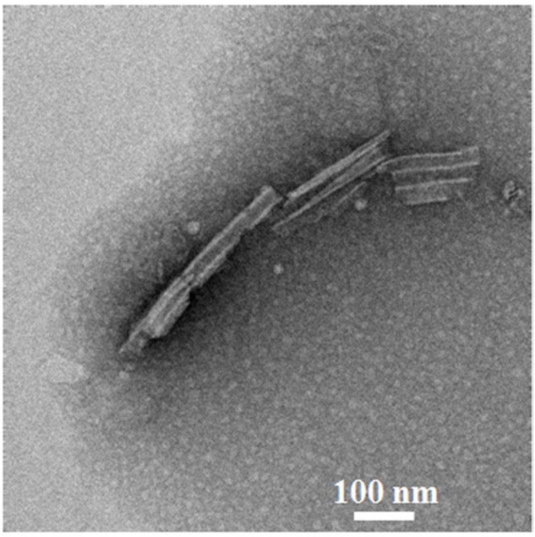

Supplementary Figure 48. TEM image of **CD-**APO2-PNTs after the adsorption of 4-aminoazobenzene.

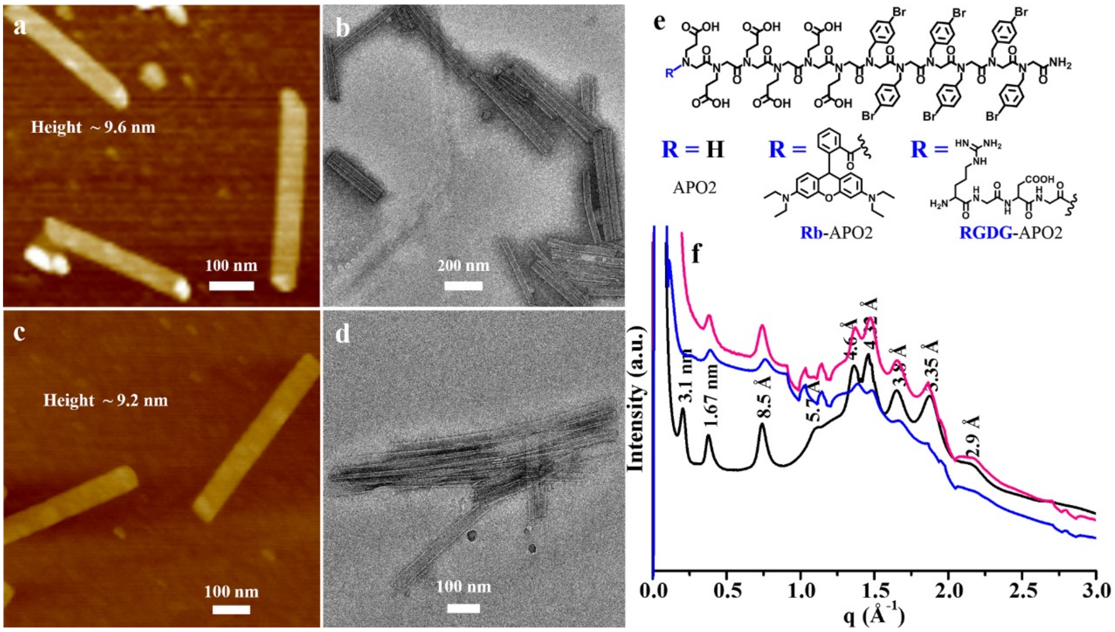

Supplementary Figure 49 | Co-assembly of two or three different APOs with to form functionalized nanotubes. (**a** and **b**) AFM and TEM images showing functionalized PNTs were co-assembled from APO2, Rb-APO2 and RGDG-APO2 with a molar ratio of 8:1:1. (**c** and **d**) AFM and TEM images showing PNTs assembled from APO2 and Rb-APO2 with a molar ratio of 9:1. (**e**) Structures of APO2, Rb-APO2 and RGDG-APO2. (**f**) XRD data of PNTs co-assembled from APO2, Rb-APO2 and RGDG-APO2 with a molar ratio of 8:1:1 (Pink), and from APO2 and Rb-APO2 with a molar ratio of 9:1 (Blue); all of these PNTs show similar XRD peaks to those of APO2-PNTs (Black), indicating they all have similar structures.
