# Supplementary material for: Designable and dynamic single-walled stiff nanotubes assembled from sequence-defined peptoids
Source: Nat Commun. 2018 Jan 18;9:270. doi: 10.1038/s41467-017-02059-1 (PMC5773689; doi:10.1038/s41467-017-02059-1)
Supplement: Supplementary file 2 — Description of Additional Supplementary Information [file 41467_2017_2059_MOESM2_ESM.pdf]

Description of Additional Supplementary Files

File Name: Supplementary Movie 1
Description: Cross-sectional view of PNT's deformation simulated by FEM.

File Name: Supplementary Movie 1
Description: 3D view of PNT's deformation simulated by FEM.
